# Incidence, recurrence, and determinants of sexually transmitted infections among people with HIV: a multicenter cohort study in China, 2010–2024, with implications for integrated HIV–STI prevention across the Western Pacific

**DOI:** 10.1016/j.lanwpc.2026.101879

**Published:** 2026-05-22

**Authors:** Xi Xiao, Liqin Sun, Yun He, Fang Zhao, Zixin Wang, Yanjun Li, Jinping Huang, Jinwei Wu, Yinsong Luo, Xiaorui Li, Chenye Liu, Dian Zhao, Yiyao Hu, Jiaye Liu, Hongzhou Lu, Ping Cen

**Affiliations:** a School of Public Health, Shenzhen University Medical School, Shenzhen, China; b Department of Infectious Diseases, National Clinical Research Center for Infectious Diseases, Shenzhen Third People's Hospital, Shenzhen, China; c JC School of Public Health and Primary Care, Faculty of Medicine, the Chinese University of Hong Kong, Hong Kong, China; d Infectious Disease Laboratory, Guangxi AIDS Clinical Treatment Center (Nanning), The Fourth People's Hospital of Nanning, Nanning, Guangxi, China

**Keywords:** Sexually transmitted infections, HIV, Epidemiology, Incidence, Recurrence

## Abstract

**Background:**

Sexually transmitted infections (STIs) remain a major public health concern among people with HIV (PWH) in the Western Pacific region, where systematic long-term surveillance is limited. As the region's largest epidemic country, China has yet to generate longitudinal evidence on STI burden, recurrence, and risk determinants, emphasizing the urgency of integrated monitoring within HIV care systems.

**Methods:**

We established a multicenter dynamic cohort including 32,961 PWH who initiated ART between 2010 and 2024 at two major HIV treatment centers in southern China. Five major STIs—syphilis, human papillomavirus (HPV), herpes simplex virus type 2 (HSV-2), gonorrhea, and chlamydia—were identified through linked clinical and laboratory records. Annual incidence rates were calculated, cumulative incidence was estimated using the Aalen–Johansen method, and demographic and clinical determinants were assessed using Fine–Gray models. Recurrence of viral infections and reinfection of bacterial STIs were identified by repeated laboratory-confirmed diagnoses during follow-up.

**Findings:**

From 2010 to 2024, 14.1% of participants experienced at least one STI after ART initiation, with the overall incidence of any STI increasing from 7.7 (95% CI 3.3–15.2) to 28.3 (95% CI 26.1–30.6) per 1000 person-years. Syphilis remained the leading infection, showing two distinct peaks in 2018 and 2024. HPV ranked second, with incidence rising steadily until 2021 before a modest decline. Gonorrhea and chlamydia resurged around 2022, while HSV-2 remained at a low but stable level. Younger age, male sex, and men who have sex with men were independent predictors of any STI. Higher baseline CD4^+^ T cell counts were associated with increased risks of syphilis and HPV, whereas HSV-2 incidence was more common in older or immunocompromised individuals. Among participants with at least one STI during the study period, 28.7% (95% CI 27.7–29.7%) experienced recurrence or reinfection, with the highest recurrence observed for HPV (33.5%, 95% CI 31.7–35.3%) and HSV-2 (29.5%, 95% CI 22.6–37.5%), while syphilis accounted for the largest number of reinfection cases (n = 1334).

**Interpretation:**

This long-term multicenter cohort provides the most comprehensive evidence to date on STI epidemiology among people with HIV in China. It reveals a persistently high burden of syphilis and HPV, a sharp resurgence of bacterial STIs, and notable recurrence of viral infections. These findings underscore the importance of strengthening comprehensive STI prevention and monitoring within HIV care settings, particularly through systematic screening and sustained surveillance efforts. By offering regionally relevant data, this study informs integrated HIV–STI control strategies and advances health equity across the Western Pacific.

**Funding:**

National Natural Science Foundation of China; the Guangxi Key Research and Development Program; the Guangdong Basic and Applied Basic Research Foundation; and the Guangdong Provincial Medical Science and Technology Research Fund Project.

**Research in context**

**Evidence before this study**

We conducted a comprehensive search of the literature using PubMed and Web of Science from inception to October 22, 2025, with the keywords “sexually transmitted infections”, “HIV”, “coinfection”, “recurrence”, “reinfection”, and “China” without language restrictions. Previous research has primarily focused on men who have sex with men (MSM) cohorts in high-income countries, often limited to single pathogens or short-term follow-up. In the Western Pacific region, which accounts for nearly one-quarter of global HIV cases, integrated surveillance of sexually transmitted infections (STIs) among people with HIV (PWH) remains scarce. China, the region's largest HIV epidemic country, lacks longitudinal multicenter evidence on STI epidemiology, recurrence patterns, and determinants in routine care settings.

**Added value of this study**

Using data from two major HIV-designated hospitals in southern China, we established a multicenter, hospital-based dynamic cohort including 32,961 adults followed between 2010 and 2024. This study provides the first long-term multicenter evidence on STI epidemiology among PWH in China, revealing a persistently high burden of syphilis and HPV, a resurgence of gonorrhea and chlamydia in recent years, and frequent recurrence of viral infections. We identified distinct risk patterns by age, sex, transmission route, and immune status—particularly elevated risks among younger adults, MSM, and those with higher baseline CD4^+^ counts—highlighting behavioral and structural drivers of ongoing transmission.

**Implications of all the available evidence**

Our findings highlight persistent syndemic interactions between HIV and STIs in the ART era, driven by immune dysregulation, behavioral adaptation, and gaps in screening and vaccination coverage. Integrating routine opt-out STI screening, HPV vaccination—including for males—and digital health–supported behavioral interventions into HIV care could substantially reduce reinfection, enhance treatment adherence, and promote health equity. This study provides regionally relevant evidence to inform HIV–STI integration frameworks and advance progress toward the WHO 2030 targets across the Western Pacific region.

## Introduction

HIV continues to pose a substantial global health burden. In 2024, approximately 40.8 million individuals were living with HIV worldwide, with 1.3 million new infections and 630,000 AIDS-related deaths, indicating that the epidemic remains far from elimination.^1^ The widespread implementation of antiretroviral therapy (ART) has transformed HIV into a chronic, manageable condition and markedly reduced mortality. However, ART cannot fully reconstitute immune function or prevent the emergence of comorbidities linked to immune activation and chronic inflammation.^2^ Furthermore, the risk of coinfections is exacerbated by shared behavioral patterns and transmission networks rather than biological vulnerability alone. Among these, sexually transmitted infections (STIs) have become a major overlapping epidemic that continues to challenge HIV prevention and care efforts.^3^

HIV and STIs form a vicious cycle. Inflammation and ulceration caused by STIs facilitate HIV transmission, whereas HIV-induced immune dysregulation and epithelial compromise increase susceptibility to new or persistent STIs.^4^, ^5^, ^6^ This bidirectional relationship perpetuates a self-sustaining cycle of transmission. Global evidence consistently shows that people with HIV (PWH) bear a disproportionately high burden of STIs.^7^ Bacterial infections such as syphilis, gonorrhea, and chlamydia have resurged even in the ART era and among virally suppressed individuals.^8^^,^^9^ For instance, global surveillance data show that syphilis incidence among men who have sex with men (MSM) increased steadily between 2015 and 2025, with a 34% rise in Europe within a single year (2021–2022)—a trend most pronounced among those living with HIV.^10^ Similar upward trends have also been observed in the Western Pacific region. In China, national surveillance data indicate that syphilis notification rates have increased by approximately 11.9% annually over the past two decades. Critically, a recent meta-analysis of nearly 35,000 Chinese PWH estimated a pooled syphilis prevalence of 19.9% (95% CI 15.4–24.8%), far exceeding that of the general population.^11^^,^^12^

STI acquisition among PWH reflects the intersection of biological recovery and behavioral adaptation. Younger adults and MSM consistently represent the most affected subgroups.^13^ In China, this disparity is particularly pronounced, with the prevalence of syphilis among HIV-positive MSM (21.9%) being more than twice that observed among heterosexual PWH (10.3%).^12^ Paradoxically, higher CD4^+^ T-cell counts following ART initiation, typically signaling immune restoration, are often linked to greater STI risk.^14^ This paradox has been attributed to renewed sexual activity and reduced risk perception, a behavioral pattern sometimes referred to as disinhibition.^15^ These trends illustrate that biomedical control alone cannot disrupt STI spread and that behavioral, social, and structural determinants remain powerful contributors. However, most existing evidence has been derived from MSM-focused cohorts in high-income settings, limiting generalizability to broader, treatment-experienced HIV populations in diverse epidemiological contexts.^16^^,^^17^

In the Western Pacific region, comprehensive longitudinal surveillance of STIs among PWH remains limited. As the region's largest epidemic country, China plays a pivotal role in informing integrated HIV–STI control strategies. Yet, despite substantial progress in ART scale-up, the existing studies remain predominantly pathogen-specific, cross-sectional, or locally confined, leaving the longitudinal incidence, recurrence patterns, and determinants of STIs insufficiently characterized in routine programmatic settings.^12^^,^^18^^,^^19^ To align with the WHO Global Health Sector Strategy (2022–2030) and strengthen integrated HIV–STI control efforts in the Western Pacific region, robust longitudinal evidence from large, programmatic cohorts is urgently needed.^20^

To address these critical gaps, we established a multicenter cohort of 32,961 adults who initiated ART between 2010 and 2024 at two major HIV care centers in southern China (Shenzhen and Nanning). Using decade-long linked clinical and laboratory data, this study evaluated the incidence, temporal trends, and determinants of five major STIs, and characterized cumulative and recurrent infection patterns. By providing the first long-term multicenter evidence from southern China in the ART era, our findings provide a robust empirical foundation to inform integrated HIV–STI prevention strategies and guide targeted interventions for high-risk populations across the Western Pacific region.

## Methods

### Study population and data sources

We conducted a multicenter, hospital-based dynamic cohort including adults who initiated ART between January 1, 2010, and September 30, 2024, at the Third People's Hospital of Shenzhen and the Fourth People's Hospital of Nanning, the designated hospitals providing long-term HIV follow-up in these two cities in China. Given the routine 3-monthly follow-up schedule for HIV care (medication refill and clinical assessment), the last eligible ART initiation date was set to September 30, 2024, to ensure that each participant had at least one scheduled follow-up. Follow-up time was calculated from the date of ART initiation (defined as time zero) until the earliest occurrence of the pathogen-specific STI event, death, loss to follow-up, or administrative censoring on December 31, 2024, whichever occurred first. Participants lost to follow-up were right-censored at the date of their last contact. For each pathogen-specific incidence analysis, individuals with documented infection at baseline were excluded from the risk set but were retained for recurrence analyses.

Participants were excluded if they (1) were younger than 18 years at ART initiation; (2) lacked essential baseline information required to define time zero or ascertain follow-up in the electronic medical record (EMR), including missing or invalid ART initiation dates; or (3) were identified during data quality control as having no documented follow-up records after ART initiation, and therefore could not contribute person-time to longitudinal analyses. Ultimately, a total of 32,961 individuals were included in the final analysis. The detailed process of participant inclusion and exclusion is illustrated in Supplementary Fig. S1. Among these participants, 3331 had a documented STI diagnosis prior to ART initiation. For analyses of incident post-ART STIs, these individuals were not considered at risk at time zero and were therefore excluded from the baseline risk set (Table 1). However, they remained eligible for analyses of STI recurrence during follow-up. For the 29,630 participants included in the incident risk set, 1215 (4.1%) were lost to follow-up before December 31, 2024, corresponding to an overall retention rate of 95.9%. Baseline and follow-up information covered demographic, clinical, and laboratory data. Data were obtained from the hospitals’ EMR systems and standardized case report forms completed during routine visits.

**Table 1:** Baseline characteristics of study participants according to STI diagnosis status.

Characteristics	Total	No STI	Any STI after ART	*P* value
N	29,630	25,466	4164	
Male, n (%)	24,245 (81.8)	20,395 (80.1)	3850 (92.5)	<0.001
Age, years, n (%)				<0.001
18–29	8766 (29.6)	6911 (27.1)	1855 (44.5)	
30–39	8478 (28.6)	7106 (27.9)	1372 (32.9)	
40–49	5283 (17.8)	4691 (18.4)	592 (14.2)	
≥50	7103 (24.0)	6758 (26.5)	345 (8.3)	
BMI, kg/m^2^, n (%)				<0.001
<18.5	5497 (18.6)	4796 (18.8)	701 (16.8)	
18.5–24.9	18,403 (62.1)	15,678 (61.6)	2725 (65.4)	
≥25.0	5730 (19.3)	4992 (19.6)	738 (17.7)	
Marital status, n (%)				<0.001
Never married	13,640 (46.0)	10,833 (42.5)	2807 (67.4)	
Married or cohabiting	12,328 (41.6)	11,288 (44.3)	1040 (25.0)	
Divorced, separated, or widowed	3564 (12.0)	3254 (12.8)	310 (7.4)	
Unknown	98 (0.3)	91 (0.4)	7 (0.2)	
HIV transmission route, n (%)				<0.001
Male-to-male sex contact	12,229 (41.3)	9431 (37.0)	2798 (67.2)	
Heterosexual contact	16,028 (54.1)	14,759 (58.0)	1269 (30.5)	
IDU	717 (2.4)	682 (2.7)	35 (0.8)	
Other	656 (2.2)	594 (2.3)	62 (1.5)	
WBC, 10ˆ9/L	5.4 ± 1.6	5.4 ± 1.7	5.5 ± 1.6	<0.001
Platelet, 10ˆ9/L	217.0 ± 63.9	217.1 ± 64.5	216.3 ± 60.0	0.432
Estimated glomerular filtration rate, mL/min/1.73 m^2^	111.4 ± 15.5	111.4 ± 15.5	115.6 ± 13.6	<0.001
Alanine aminotransferase, U/L	23.1 ± 12.0	23.0 ± 12.1	23.2 ± 11.7	0.376
Aspartate aminotransferase, U/L	24.3 ± 8.0	24.4 ± 8.1	23.7 ± 7.5	<0.001
Hypercholesterolemia, n (%)	2188 (7.4)	2037 (8.0)	151 (3.6)	<0.001
Hypertension, n (%)	943 (3.2)	886 (3.5)	57 (1.4)	<0.001
CKD, n (%)	749 (2.5)	696 (2.7)	53 (1.3)	<0.001
Diabetes, n (%)	1449 (4.9)	1358 (5.3)	91 (2.2)	<0.001
HBV infection, n (%)	3239 (10.9)	2812 (11.0)	427 (10.3)	0.117
HCV infection, n (%)	912 (3.1)	842 (3.3)	70 (1.7)	<0.001
Opportunistic infections, n (%)	1034 (3.5)	871 (3.4)	163 (3.9)	0.107
Time interval, months, n (%)				<0.001
<1	17,521 (59.1)	15,221 (59.8)	2300 (55.2)	
≥1	12,109 (40.9)	10,245 (40.2)	1864 (44.8)	
Initial CD4 count, cells/μL, n (%)				<0.001
<200	12,227 (41.3)	10,753 (42.2)	1474 (35.4)	
200–349	9854 (33.3)	8367 (32.9)	1487 (35.7)	
≥350	7549 (25.5)	6346 (24.9)	1203 (28.9)	
Initial CD8 count, cells/μL, n (%)				<0.001
<500	5965 (20.1)	5371 (21.1)	594 (14.3)	
500–999	13,057 (44.1)	11,171 (43.9)	1886 (45.3)	
≥1000	10,608 (35.8)	8924 (35.0)	1684 (40.4)	
Initial HIV RNA, copies/mL, n (%)				<0.001
<100,000	13,780 (46.5)	11,954 (46.9)	1826 (43.9)	
≥100,000	15,850 (53.5)	13,512 (53.1)	2338 (56.1)	
Types of incident STIs after ART, n (%)				<0.001
Syphilis	2681 (9.0)	0 (0.0)	2681 (64.4)	
HPV	1768 (6.0)	0 (0.0)	1768 (42.5)	
HSV-2	78 (0.3)	0 (0.0)	78 (1.9)	
Gonorrhea	59 (0.2)	0 (0.0)	59 (1.4)	
Chlamydia	71 (0.2)	0 (0.0)	71 (1.7)	
ART treatment regimen, n (%)				<0.001
2NRTIs + EFV	20,568 (69.4)	17,443 (68.5)	3125 (75.0)	
2NRTIs + LPV/r	2113 (7.1)	1903 (7.5)	210 (5.0)	
BIC/FTC/TAF	1697 (5.7)	1518 (6.0)	179 (4.3)	
2NRTIs + NVP	1817 (6.1)	1582 (6.2)	235 (5.6)	
DTG-containing	1432 (4.8)	1279 (5.0)	153 (3.7)	
EVG/c/FTC/TAF	648 (2.2)	523 (2.1)	125 (3.0)	
3TC + LPV/r	642 (2.2)	601 (2.4)	41 (1.0)	
2NRTIs + ANV/DOR	256 (0.9)	235 (0.9)	21 (0.5)	
Others	457 (1.5)	382 (1.5)	75 (1.8)	

Continuous variables are expressed as mean ± standard deviation (SD), and comparisons between groups were performed using the t test. Categorical variables are presented as frequency (%) and compared using Pearson's χ^2^ test. Time interval refers to the duration between HIV diagnosis and ART initiation. STI types are not mutually exclusive.

STI, sexually transmitted infection; ART, antiretroviral therapy; BMI, body-mass index; HIV, human immunodeficiency virus; IDU, injection drug use; WBC, white blood cells; CKD, chronic kidney disease; HBV, hepatitis B virus; HCV, hepatitis C virus; HPV, human papillomavirus; HSV-2, herpes simplex virus type 2; NRTIs, nucleoside reverse transcriptase inhibitors; ANV, ainuovirine; NVP, nevirapine; LPV/r, lopinavir/ritonavir; EFV, efavirenz; DOR, doravirine; BIC, bictegravir; FTC, emtricitabine; TAF, tenofovir alafenamide; EVG, elvitegravir; DTG, dolutegravir.

### Assessment of covariates

All data were extracted from the EMR systems and updated during follow-up: (1) demographic characteristics, including age at HIV diagnosis (18–29, 30–39, 40–49, and ≥50 years), sex assigned at birth, marital status (never married, married or cohabiting, divorced or separated, widowed, or unknown), and body mass index (BMI, <18.5, 18.5–24.9, and ≥25.0 kg/m^2^); (2) laboratory parameters, such as white blood cell (WBC) count, platelet (PLT) count, estimated glomerular filtration rate (eGFR), alanine aminotransferase (ALT), aspartate aminotransferase (AST); (3) HIV-related factors, including route of HIV transmission (male-to-male sexual contact, heterosexual contact, injection drug use, or other), CD4^+^ and CD8^+^ T-cell count at ART initiation, initial plasma HIV RNA levels, ART treatment regimen at initiation, time interval between HIV diagnosis and ART initiation, and the presence of opportunistic infections; (4) comorbidities, including diabetes (defined as fasting plasma glucose ≥7.0 mmol/L, prescription of antidiabetic medication, or self-reported diabetes), chronic kidney disease (CKD, defined as eGFR<60 mL/min/1.73 m^2^ sustained for ≥3 months or clinical diagnosis of CKD), hepatitis B virus (HBV) infection (defined as positive hepatitis B surface antigen (HBsAg) test or detectable HBV DNA), hepatitis C virus (HCV) infection (defined as detectable HCV RNA, indicating active infection), hypercholesterolemia (defined by a total cholesterol level of ≥5.2 mmol/L or use of lipid-lowering agents), and hypertension (defined as systolic blood pressure ≥140 mmHg or diastolic blood pressure ≥90 mmHg). Race and ethnicity data were not collected in the routine clinical systems of the participating hospitals and were therefore unavailable for analysis.

### Follow-up and outcomes

The primary outcome was the incidence of the first diagnosis of any of the five predefined STIs after ART initiation, including syphilis, human papillomavirus (HPV), herpes simplex virus type 2 (HSV-2), gonorrhea, and chlamydia. Participants were considered to have an incident STI if they were diagnosed with any of these infections after starting ART. The event date was defined as the actual calendar date of laboratory confirmation recorded in the electronic medical record, rather than the date of a scheduled visit. Participants attended routine HIV follow-up approximately every 3 months; however, STI testing was not limited to these visits and could be performed whenever clinically indicated. Accordingly, diagnosis dates reflected the calendar time of laboratory confirmation and were analyzed under a right-censored time-to-event framework. Person-time at risk was calculated from the date of ART initiation to the date of the first STI diagnosis, death, or the end of the study period (December 31, 2024), whichever occurred first. Annual incidence rates were expressed as the number of new cases per 1000 person-years, calculated as the number of incident cases divided by total person-time at risk and multiplying by 1000.

STI outcomes were ascertained using pathogen-specific laboratory diagnostic procedures consistent with internationally recognized clinical guidelines.^21^ These procedures were performed during scheduled follow-up visits and unscheduled clinical encounters. Syphilis was diagnosed through combined treponemal and non-treponemal serologic testing. Gonorrhea and chlamydia were identified by nucleic acid amplification tests (NAATs) performed on appropriate clinical specimens, with culture-based confirmation used where available. HPV infection was defined by detection of HPV DNA using polymerase chain reaction (PCR)-based genotyping assays. HSV-2 infection was determined by type-specific (glycoprotein G–based) serology and/or PCR testing of lesion specimens. Diagnostic platforms, interpretation thresholds, and case ascertainment procedures were harmonized across the two participating centers to ensure methodological consistency throughout the study period. STI testing was performed based on clinical indications, patient-reported symptoms, or clinician assessment, rather than systematic universal screening. Further diagnostic details are provided in Supplementary Table S1.

Secondary outcomes included pathogen-specific incidence rates, annual temporal trends, subgroup differences by demographic and clinical characteristics, cumulative infection patterns, and subsequent STI episodes during follow-up. Recurrent events were categorized as reinfections for bacterial pathogens (syphilis, gonorrhea, and chlamydia) and recurrences for viral pathogens (HPV and HSV-2), following diagnostic standards from the US Centers for Disease Control and Prevention (CDC) 2021 STI Guidelines.^21^ Specifically, reinfection for bacterial STIs was defined by a ≥ 4-fold rise in non-treponemal titers for syphilis, repeat NAAT or culture positivity following documented completion of recommended therapy for gonorrhea, with clinical assessment used to distinguish reinfection from treatment failure, and repeat NAAT positivity after documented treatment completion for chlamydia, with review of clinical history to exclude persistent infection. Meanwhile, recurrence for viral STIs was defined by repeat laboratory-confirmed detection (PCR or DNA testing) following prior clinical resolution or documented negative testing, consistent with CDC-recommended diagnostic frameworks. Detailed information is summarized in Supplementary Table S2.

### Statistical analysis

All analyses were performed using R software (version 4.4.0; https://www.r-project.org). Continuous variables are expressed as mean ± standard deviation (SD), and comparisons between groups were performed using the t-test. Categorical variables were presented as counts (percentages) and compared using the χ^2^ test. Baseline characteristics were compared across participants eligible for post-ART incident STI analyses, with baseline uniformly defined as the time of ART initiation. For time-to-event analyses, participants were further classified by the occurrence of an incident post-ART STI during follow-up (yes vs no). All statistical tests were two-sided, with *P* value <0.05 was considered statistically significant.

Incidence rates of any STI and each specific infection were calculated per 1000 person-years, with 95% confidence intervals (CIs) estimated under a Poisson distribution. Because death could preclude the occurrence of STIs during follow-up, it was treated as a competing event rather than as censoring. Cumulative incidence functions for the first STI and each specific infection were estimated using the Aalen–Johansen method. Group differences were assessed using Gray's test. Cumulative incidence curves were stratified by four key subgroups: age, sex, CD4^+^ T-cell count at ART initiation, and route of HIV transmission.

To identify predictors of incident STIs while accounting for death as a competing event, Fine–Gray subdistribution hazard regression models were constructed. Multivariable models included all covariates described in the “Assessment of covariates” section, encompassing demographic characteristics (age, sex, marital status, and BMI), laboratory parameters (WBC, PLT, eGFR, ALT, AST), HIV-related factors (route of transmission, CD4^+^ T-cell and CD8^+^ T-cell count at ART initiation, baseline plasma HIV RNA level, ART regimen at initiation, and interval between HIV diagnosis and ART initiation), and comorbidities (diabetes, CKD, HBV infection, HCV infection, hypercholesterolemia, hypertension, and opportunistic infections). Covariates were selected a priori based on clinical relevance and prior evidence. Given that our goal was association modeling across multiple baseline predictors rather than estimation of a causal effect of a single treatment, we did not implement propensity score matching or weighting. Results were reported as subdistribution hazard ratios (sHRs) with 95% confidence intervals (CIs). The PSH assumption was assessed using a modified weighted Schoenfeld residual–based score test, complemented by time-varying sHR(t) diagnostics (Supplementary Fig. S2–S7). Given evidence that the proportional subdistribution hazards (PSH) assumption was not met, we used time-stratified (piecewise) Fine–Gray models as the primary analytic approach, allowing covariate effects to vary across prespecified follow-up windows. Follow-up time was partitioned into 0–1 year, 1–3 years, and >3 years (to an outcome-specific administrative censoring time, τ), with cut-points informed by the empirical distribution of event times (higher early event density) and clinical considerations regarding early post-ART risk dynamics and subsequent stabilization. Time-stratified sHRs were reported for each interval. Missing covariate data (<5% for all variables) were handled using multiple imputation by chained equations. As a sensitivity analysis, we recalculated annual STI incidence rates after excluding participants with only one follow-up visit. Sensitivity analyses comparing complete-case and imputed models yielded consistent results.

### Ethics approval

The study adhered to the ethical guidelines of the 1975 Declaration of Helsinki and was approved by the Institutional Review Board of Shenzhen University (PN-202500096). This study used deidentified routinely collected clinical data, and the requirement for written informed consent was waived in accordance with institutional review board policies and applicable national regulations.

### Role of funding source

The funders of the study had no role in the study design, data collection, data analysis, data interpretation, writing of the report, or the decision to submit the manuscript for publication.

## Results

### Participant characteristics

Among the 32,961 participants included in the overall cohort, 3331 (10.1%) had a documented STI diagnosis prior to ART initiation. These individuals were excluded from the post-ART incident risk set and were included in subsequent analyses of cumulative infection patterns and recurrence (Supplementary Table S3).

Among the remaining 29,630 PWH who initiated ART between 2010 and 2024 and were included in the post-ART incident risk set, 4164 (14.1%) developed at least one STI during follow-up. Among participants with post-ART STIs, syphilis accounted for 64.4% of infections, followed by HPV (42.5%), HSV-2 (1.9%), gonorrhea (1.4%), and chlamydia (1.7%) (*P* < 0.001). Baseline characteristics at ART initiation are presented in Table 1 according to subsequent incident STI status.

Participants who developed incident STIs were more frequently male (92.5% vs 80.1%; *P* < 0.001), younger (44.5% aged 18–29 years vs 27.1% in the no-STI group; *P* < 0.001), and never married (67.4% vs 42.5%; *P* < 0.001). Male-to-male sexual contact was substantially more common among those who developed incident STIs (67.2% vs 37.0%), whereas heterosexual transmission predominated in the no-STI group (58.0% vs 30.5%; *P* < 0.001). Baseline immunological profiles differed between groups. Participants who subsequently developed incident STIs were more likely to have CD4^+^ T-cell counts ≥350 cells/μL (28.9% vs 24.9%) and less likely to have CD4^+^ T-cell counts <200 cells/μL (35.4% vs 42.2%; *P* < 0.001). A similar pattern was observed for CD8 counts, with higher proportions of CD8^+^ T-cell ≥1000 cells/μL in the incident STI group (40.4% vs 35.0%; *P* < 0.001). Baseline HIV RNA levels were also modestly higher among participants who developed incident STIs (56.1% vs 53.1% with ≥100,000 copies/mL; *P* < 0.001). Regarding laboratory parameters, estimated glomerular filtration rate was higher in the incident STI group (115.6 vs 111.4 mL/min/1.73 m^2^; *P* < 0.001), while AST levels were slightly lower. WBC counts were marginally higher, whereas platelet and ALT levels did not differ significantly between groups.

Regarding comorbidities, hypercholesterolemia, hypertension, CKD, diabetes, and HCV infection were less prevalent among participants who subsequently developed incident STIs (all *P* < 0.001), while HBV infection and opportunistic infections did not differ significantly between groups. Initial ART regimens also varied across groups (*P* < 0.001).

### STI incidence temporal trends

**Fig. 1:**
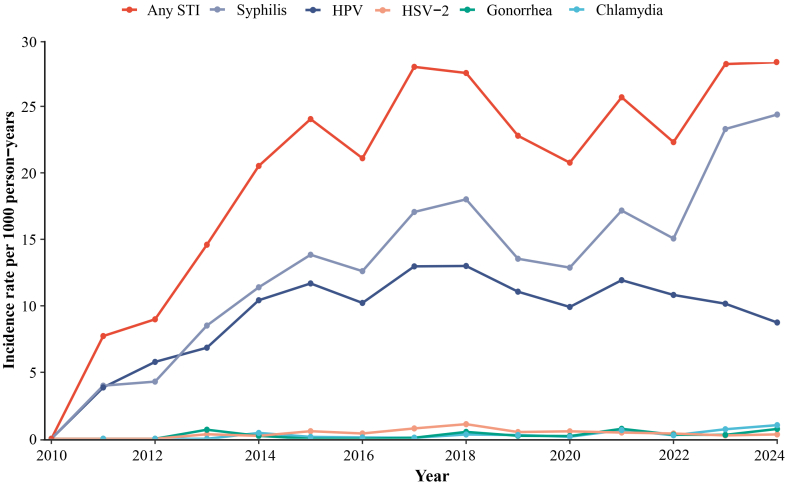
Temporal trends in annual incidence rates of any STI, syphilis, HPV, HSV-2, gonorrhea, and chlamydia among PWH, 2010–2024. STI, sexually transmitted infection; HPV, human papillomavirus; HSV-2, herpes simplex virus type 2; PWH, people with HIV.

Between 2010 and 2024, the incidence rate of any STI among PWH showed a marked upward trajectory, increasing from 7.7 (95% CI 3.3–15.2) to 28.3 (26.1–30.6) per 1000 person-years (Fig. 1). Syphilis remained the predominant infection throughout the study period, with its incidence rising sharply from 4.0 (95% CI 1.1–10.2) per 1000 person-years in 2011 to 18.0 (15.8–20.4) in 2018, followed by a secondary surge to 24.4 (22.3–26.6) by 2024. HPV ranked second, increasing steadily from 3.9 (95% CI 1.1–9.9) per 1000 person-years in 2011 to a peak of 11.9 (10.5–13.5) in 2021, before declining slightly thereafter. By contrast, the incidence of the other three STIs remained relatively low. HSV-2 fluctuated modestly between 0.3 (95% CI 0.1–1.9) and 1.1 (0.6–1.8) per 1000 person-years throughout the study period. Gonorrhea showed intermittent fluctuations, peaking at 0.8 (95% CI 0.4–1.2) per 1000 person-years in 2021, dropping by nearly half in 2022, and returning to a similar level by 2024. Chlamydia increased gradually from 0.4 (95% CI 0.1–1.6) to 1.0 (0.7–1.5) per 1000 person-years by 2024, eventually surpassing both HSV-2 and gonorrhea in the later years of follow-up. A sensitivity analysis excluding participants with only one follow-up visit produced highly consistent annual incidence estimates and temporal trends (Supplementary Fig. S8).

### Aalen–Johansen cumulative incidence of STIs

**Fig. 2:**
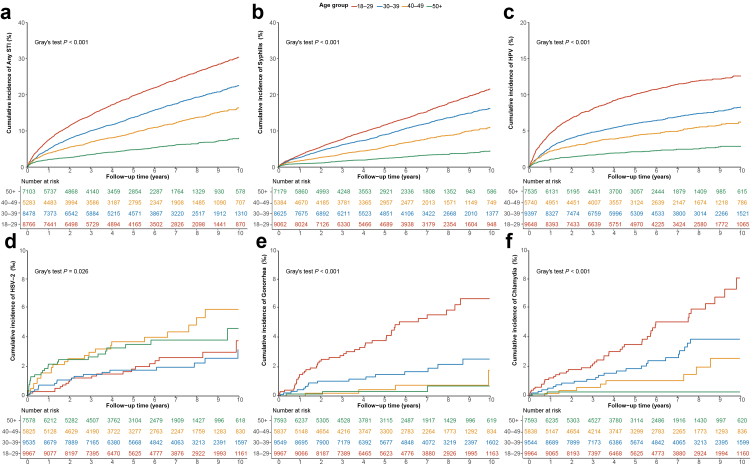
Aalen–Johansen cumulative incidence curves for any STI, syphilis, HPV, HSV-2, gonorrhea, and chlamydia, stratified by age group. Panels a–f show the cumulative incidence curves for any STI, syphilis, HPV, HSV-2, gonorrhea, and chlamydia, respectively. STI, sexually transmitted infection; HPV, human papillomavirus; HSV-2, herpes simplex virus type 2.

**Fig. 3:**
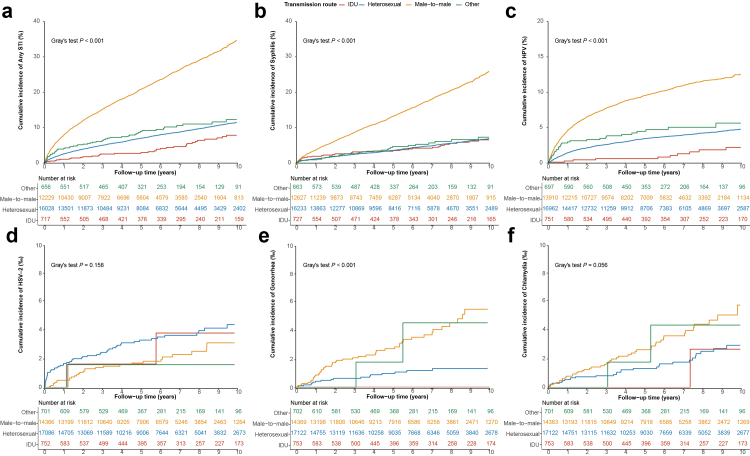
Aalen–Johansen cumulative incidence curves for any STI, syphilis, HPV, HSV-2, gonorrhea, and chlamydia, stratified by HIV transmission route. Panels a–f show the cumulative incidence curves for any STI, syphilis, HPV, HSV-2, gonorrhea, and chlamydia, respectively. STI, sexually transmitted infection; HPV, human papillomavirus; HSV-2, herpes simplex virus type 2; IDU, injection drug use.

Aalen–Johansen analyses demonstrated distinct cumulative incidence patterns of STIs across demographic, behavioral, and immunologic subgroups. Age-stratified analyses revealed a clear gradient for most infections. Younger adults aged 18–29 years had the highest cumulative risks of any STI, syphilis, gonorrhea, chlamydia and HPV, with steadily lower incidence observed in older age groups (Gray's test *P* < 0.001) (Fig. 2). In contrast, HSV-2 exhibited an atypical pattern, as participants aged 40 years or older showed higher cumulative incidence than younger individuals. By HIV transmission route, MSM had the greatest overall burden, with markedly higher cumulative incidence of any STI, syphilis, and HPV than heterosexuals, injection drug users, or others (Gray's test *P* < 0.001) (Fig. 3). Gonorrhea incidence also remained highest among MSM with statistically significant between-group differences (Gray's test *P* < 0.001), whereas chlamydia demonstrated more modest variation across exposure categories and did not reach statistical significance. HSV-2 incidence varied comparatively little by transmission route. Sex-specific analyses indicated that men carried higher cumulative risks than women for any STI, syphilis, HPV, and gonorrhea (Gray's test *P* < 0.001) (Supplementary Fig. S9). In contrast, cumulative incidence of chlamydia and HSV-2 did not differ significantly between sexes, although women tended to experience higher chlamydia incidence during much of follow-up. Participants with baseline CD4 ≥ 350 cells/μL consistently showed higher cumulative incidence of any STI, syphilis, and HPV (Gray's test *P* < 0.05), whereas HSV-2 diverged, with incidence being higher among those with CD4 < 350 cells/μL (Supplementary Fig. S10). No statistically significant differences across CD4 strata were observed for gonorrhea or chlamydia.

### Cumulative patterns of STI infections among PWH, 2010–2024

**Fig. 4:**
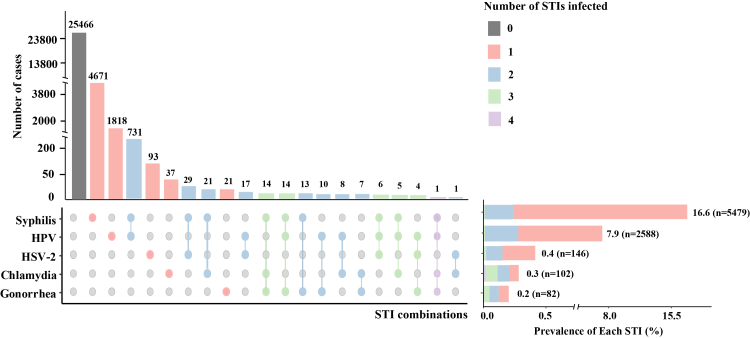
Cumulative patterns of multiple STIs among PWH, 2010–2024. STIs, sexually transmitted infections; HPV, human papillomavirus; HSV-2, herpes simplex virus type 2; PWH, people with HIV.

The UpSet analysis illustrated the cumulative patterns of STIs among PWH between 2010 and 2024 (Fig. 4). Overall, 22.7% of participants experienced at least one STI during the study period, including 20.0% with a single infection and 2.7% with multiple infections. Syphilis was the most frequently reported infection (n = 5479; 16.6%), followed by HPV (n = 2588; 7.9%). HSV-2, chlamydia, and gonorrhea accounted for 0.4%, 0.3%, and 0.2% of all infections, respectively. Among participants with multiple infections, dual infections were most common (n = 843; 2.6%), primarily involving syphilis and HPV (n = 731). Triple or quadruple infections were rare (<0.5% of all participants) and typically consisted of syphilis–chlamydia–gonorrhea or syphilis–HPV–gonorrhea combinations.

### Time-stratified competing-risk regression of factors associated with incident STIs

Because the proportional subdistribution hazards assumption was not met, we reported time-stratified sHRs from fully adjusted Fine–Gray competing-risk models across three follow-up windows (0–1 year, 1–3 years, and >3 years to τ; outcome-specific administrative censoring). Across outcomes, a coherent risk profile emerged, characterized by younger age, MSM status, male sex, and lower baseline CD4 counts (Table 2).

**Table 2:** Time–stratified subdistribution hazard ratios (sHRs) from fully adjusted Fine–Gray models.

Characteristics	0–1 year		1–3 years		3–τ years	
	sHR (95% CI)	*P*	sHR (95% CI)	*P*	sHR (95% CI)	*P*
**Any STI (τ = 9)**						
Age (years)						
≥50	1.00 (reference)		1.00 (reference)		1.00 (reference)	
18–29	1.72 (1.36–2.19)	<0.001	2.33 (1.70–3.18)	<0.001	1.75 (1.37–2.25)	<0.001
30–39	1.35 (1.08–1.70)	0.009	2.13 (1.58–2.86)	<0.001	1.67 (1.32–2.12)	<0.001
40–49	1.38 (1.10–1.73)	0.006	1.65 (1.21–2.24)	0.001	1.57 (1.24–1.99)	<0.001
Sex						
Female	1.00 (reference)		1.00 (reference)		1.00 (reference)	
Male	1.49 (1.18–1.88)	<0.001	2.10 (1.56–2.81)	<0.001	1.38 (1.12–1.71)	0.003
HIV transmission route						
All other transmission routes	1.00 (reference)		1.00 (reference)		1.00 (reference)	
Male-to-male sex contact	2.03 (1.74–2.37)	<0.001	1.71 (1.45–2.02)	<0.001	1.95 (1.68–2.26)	<0.001
Initial CD4 count (cells/μL)						
≥350	1.00 (reference)		1.00 (reference)		1.00 (reference)	
200–349	0.89 (0.78–1.02)	0.099	0.91 (0.78–1.06)	0.214	0.90 (0.79–1.02)	0.113
<200	1.05 (0.91–1.22)	0.506	0.97 (0.82–1.15)	0.712	0.80 (0.69–0.94)	0.005
**Syphilis (τ** = **9)**						
Age (years)						
≥50	1.00 (reference)		1.00 (reference)		1.00 (reference)	
18–29	1.42 (0.98–2.06)	0.060	2.69 (1.76–4.11)	<0.001	1.95 (1.44–2.64)	<0.001
30–39	1.45 (1.03–2.05)	0.033	2.67 (1.78–3.99)	<0.001	1.91 (1.44–2.55)	<0.001
40–49	1.30 (0.93–1.82)	0.129	1.87 (1.24–2.82)	0.003	1.76 (1.33–2.34)	<0.001
Sex						
Female	1.00 (reference)		1.00 (reference)		1.00 (reference)	
Male	2.17 (1.48–3.17)	<0.001	3.79 (2.36–6.08)	<0.001	2.76 (2.03–3.76)	<0.001
HIV transmission route						
All other transmission routes	1.00 (reference)		1.00 (reference)		1.00 (reference)	
Male-to-male sex contact	1.75 (1.40–2.19)	<0.001	1.88 (1.52–2.32)	<0.001	1.99 (1.68–2.34)	<0.001
Initial CD4 count (cells/μL)						
≥350	1.00 (reference)		1.00 (reference)		1.00 (reference)	
200–349	0.80 (0.66–0.96)	0.018	0.89 (0.75–1.06)	0.205	0.89 (0.78–1.03)	0.116
<200	0.81 (0.65–1.02)	0.068	0.77 (0.62–0.95)	0.015	0.74 (0.62–0.88)	<0.001
**HPV (τ** = **7)**						
Age (years)						
≥50	1.00 (reference)		1.00 (reference)		1.00 (reference)	
18–29	2.23 (1.63–3.03)		2.39 (1.54–3.69)		2.16 (1.36–3.44)	<0.001
30–39	1.49 (1.11–2.01)		1.84 (1.22–2.79)		1.61 (1.03–2.52)	<0.001
40–49	1.58 (1.17–2.14)		1.48 (0.95–2.30)		1.52 (0.96–2.42)	<0.001
Sex						
Female	1.00 (reference)		1.00 (reference)		1.00 (reference)	
Male	1.31 (0.97–1.77)	0.076	1.32 (0.90–1.92)	0.152	0.69 (0.49–0.97)	0.035
HIV transmission route						
All other transmission routes	1.00 (reference)		1.00 (reference)		1.00 (reference)	
Male-to-male sex contact	2.04 (1.69–2.47)	<0.001	1.60 (1.27–2.03)	<0.001	1.68 (1.27–2.23)	<0.001
Initial CD4 count (cells/μL)						
≥350	1.00 (reference)		1.00 (reference)		1.00 (reference)	
200–349	1.00 (0.84–1.19)	0.992	0.98 (0.79–1.23)	0.886	0.97 (0.75–1.25)	0.814
<200	1.19 (0.99–1.43)	0.072	1.27 (1.00–1.61)	0.053	1.09 (0.83–1.43)	0.534
**HSV–2 (τ** = **8.5)**						
Age (years)						
≥50	1.00 (reference)		1.00 (reference)		1.00 (reference)	
18–29	0.66 (0.10–4.29)	0.665	1.76 (0.31–9.95)	0.523	1.24 (0.24–6.54)	0.799
30–39	0.74 (0.28–1.94)	0.543	1.64 (0.32–8.26)	0.551	0.86 (0.20–3.72)	0.836
40–49	0.97 (0.42–2.21)	0.939	3.53 (0.84–14.82)	0.084	2.80 (0.69–11.28)	0.149
Sex						
Female	1.00 (reference)		1.00 (reference)		1.00 (reference)	
Male	1.38 (0.59–3.24)	0.461	5.17 (0.61–44.14)	0.133	1.54 (0.50–4.79)	0.454
HIV transmission route						
All other transmission routes	1.00 (reference)		1.00 (reference)		1.00 (reference)	
Male-to-male sex contact	0.78 (0.32–1.88)	0.583	0.77 (0.29–2.03)	0.594	0.50 (0.21–1.17)	0.109
Initial CD4 count (cells/μL)						
≥350	1.00 (reference)		1.00 (reference)		1.00 (reference)	
200–349	1.63 (0.31–8.49)	0.560	2.99 (0.86–10.37)	0.084	0.94 (0.39–2.29)	0.889
<200	6.22 (1.37–28.17)	0.018	2.21 (0.59–8.27)	0.238	0.77 (0.29–2.06)	0.604
**Gonorrhea (τ** = **8)**						
Age (years)						
≥50	1.00 (reference)		1.00 (reference)		1.00 (reference)	
18–29	3.19 (0.33–30.49)	0.315	6.27 (0.23–170.48)	0.276	21.96 (2.34–206.08)	0.007
30–39	1.17 (0.11–12.46)	0.899	3.20 (0.13–80.79)	0.48	6.20 (0.80–48.27)	0.081
40–49	–	–	1.04 (0.03–31.44)	0.982	2.60 (0.25–27.60)	0.427
Sex						
Female	1.00 (reference)		1.00 (reference)		1.00 (reference)	
Male	2.33 (0.18–30.00)	0.516	3.42 (1.13–10.32)	<0.001	14.8 (1.47–89.62)	<0.001
HIV transmission route						
All other transmission routes	1.00 (reference)		1.00 (reference)		1.00 (reference)	
Male-to-male sex contact	0.69 (0.22–2.11)	0.514	2.62 (0.49–14.10)	0.261	1.27 (0.47–3.39)	0.639
Initial CD4 count (cells/μL)						
≥350	1.00 (reference)		1.00 (reference)		1.00 (reference)	
200–349	0.72 (0.25–2.07)	0.548	0.40 (0.12–1.30)	0.129	2.10 (0.69–6.38)	0.191
<200	0.43 (0.11–1.72)	0.231	0.92 (0.35–2.41)	0.862	2.06 (0.60–7.09)	0.250
**Chlamydia (τ** = **8.5)**						
Age (years)						
≥50	1.00 (reference)		1.00 (reference)		1.00 (reference)	
18–29	4.49 (1.17–17.22)	0.028	10.47 (1.73–59.05)	<0.001	24.56 (7.80.11–77.24)	<0.001
30–39	2.60 (0.68–9.98)	0.163	6.56 (1.65–26.14)	<0.001	17.13 (6.92–42.42)	<0.001
40–49	1.23 (0.16–9.63)	0.844	3.28 (0.62–17.24)	<0.001	11.12 (3.99–30.94)	<0.001
Sex						
Female	1.00 (reference)		1.00 (reference)		1.00 (reference)	
Male	0.05 (0.01–0.40)	0.005	1.37 (0.14–13.54)	0.786	0.49 (0.17–1.41)	0.187
HIV transmission route						
All other transmission routes	1.00 (reference)		1.00 (reference)		1.00 (reference)	
Male-to-male sex contact	3.80 (0.50–28.64)	0.195	1.66 (0.34–7.99)	0.528	0.81 (0.37–1.80)	0.610
Initial CD4 count (cells/μL)						
≥350	1.00 (reference)		1.00 (reference)		1.00 (reference)	
200–349	0.91 (0.34–2.41)	0.849	0.99 (0.24–4.05)	0.993	1.22 (0.52–2.86)	0.643
<200	0.19 (0.03–1.10)	0.064	0.95 (0.25–3.67)	0.941	1.23 (0.48–3.15)	0.670

Time windows were defined as 0–1 year, 1–3 years, and 3–τ years since antiretroviral therapy initiation, where τ denotes the outcome-specific administrative censoring time (τ shown for each outcome). Each cell reports the subdistribution hazard ratio with 95% confidence interval and the corresponding P value. The table presents selected covariates. Fine–Gray models treated death as a competing event and were fully adjusted for the covariates specified in the “Assessment of covariates” section, including demographic characteristics (age, sex, marital status, and body-mass index), laboratory parameters (white blood cell count, platelet count, estimated glomerular filtration rate, alanine aminotransferase, and aspartate aminotransferase), HIV-related factors (route of transmission, CD4+ T-cell count and CD8+ T-cell count at antiretroviral therapy initiation, baseline plasma HIV RNA level, ART treatment regimen at initiation, and the interval between HIV diagnosis and antiretroviral therapy initiation), and comorbidities (diabetes, chronic kidney disease, hepatitis B virus infection, hepatitis C virus infection, hypercholesterolemia, hypertension, and opportunistic infections). For parsimony, HIV transmission route was collapsed into male-to-male sex contact versus all other transmission routes. Outcome-specific τ was implemented via administrative censoring.

STI, sexually transmitted infection; HPV, human papillomavirus; HSV-2, herpes simplex virus type 2.

Age showed the most consistent gradient. Compared with participants aged ≥50 years, those aged 18–29 years had persistently higher risk of any STI across windows (0–1 year: sHR = 1.72, 95% CI 1.36–2.19; *P* < 0.001; 1–3 years: sHR = 2.33, 95% CI 1.70–3.18; *P* < 0.001; >3 years: sHR = 1.75, 95% CI 1.37–2.25; *P* < 0.001; Table 2). MSM status was similarly robust for any STI (all *P* < 0.001 across windows). Male sex was independently associated with increased risk of any STI, with the strongest association during 1–3 years (sHR = 2.10, 95% CI 1.56–2.81; *P* < 0.001).

These patterns were broadly mirrored in pathogen-specific outcomes (Table 2). For syphilis, male sex and MSM showed large and consistent effects across windows (all *P* < 0.001), and the excess risk associated with younger age became most evident after the first year (18–29 vs ≥ 50 years at 1–3 years: sHR = 2.69, 95% CI 1.76–4.11; *P* < 0.001). For HPV, younger age and MSM remained associated with higher incidence across follow-up. Immune parameters showed outcome-specific heterogeneity; notably, CD4 <200 cells/μL predicted increased HSV-2 incidence within the first year (sHR = 6.22, 95% CI 1.37–28.17; *P* = 0.018). For gonorrhea and chlamydia, sparse events within time windows resulted in imprecise and occasionally unstable estimates; these results were considered exploratory.

Associations for the remaining fully adjusted covariates, including demographic, laboratory, HIV-related, and comorbidity variables, are presented in Supplementary Table S4.

### Recurrence and reinfection of STIs

**Fig. 5:**
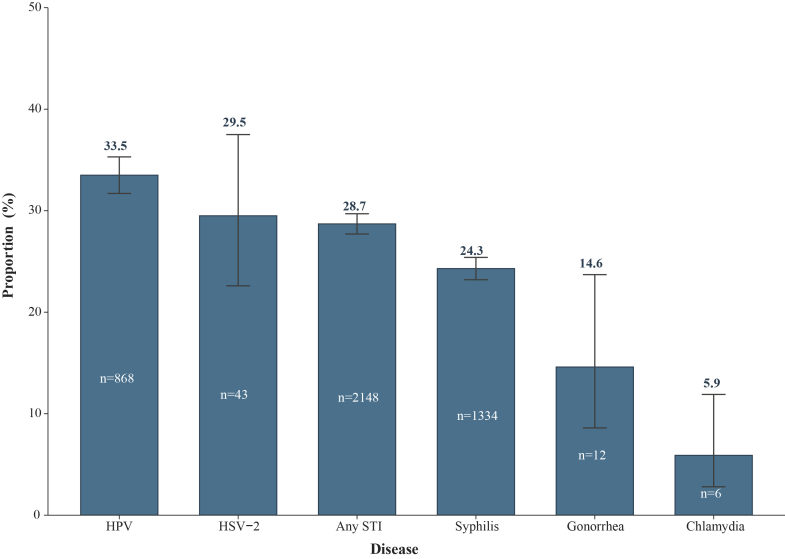
Proportions of STI recurrence and reinfection during 2010–2024. STIs, sexually transmitted infections; HPV, human papillomavirus; HSV-2, herpes simplex virus type 2.

Among participants who had ever been diagnosed with at least one STI during the study period (n = 7495), 2148 individuals (28.7%, 95% CI 27.7–29.7%) experienced a recurrence or reinfection of any STI (Fig. 5). The overall burden of recurrence was dominated by viral infections. HPV exhibited the highest recurrence rate, with 868 recurrent episodes, accounting for 33.5% (95% CI 31.7–35.3%) of all HPV cases. HSV-2 showed a similarly high recurrence burden, with 43 recurrent episodes (29.5%, 95% CI 22.6–37.5%). For bacterial infections, syphilis contributed to the largest number of reinfections, with 1334 recurrent diagnoses corresponding to 24.3% (95% CI 23.2–25.4%) of all syphilis cases. In contrast, reinfections were relatively infrequent for gonorrhea (12 cases; 14.6%, 95% CI 8.6–23.7%) and chlamydia (6 cases; 5.9%, 95% CI 2.8–11.9%).

## Discussion

This large multicenter cohort study provides novel and comprehensive insights into the long-term epidemiology of STIs among PWH in China. Despite the widespread ART coverage, STIs continue to represent a substantial public health burden, with syphilis and HPV remaining the most prevalent infections and gonorrhea and chlamydia showing a marked resurgence in recent years. Younger age, male sex, and MSM transmission were independently associated with an increased risk of acquiring STI. Associations with baseline CD4^+^ T-cell counts varied by pathogen, whereas HSV-2 incidence was higher among older adults and those with lower CD4^+^ T-cell counts. Collectively, these findings underscore the persistent vulnerability of PWH to STIs in the ART era and provide robust evidence to guide integrated screening, prevention, and behavioral intervention strategies tailored to high-risk populations.

The sustained predominance of syphilis and HPV in our cohort, despite widespread ART coverage, reflects both regional transmission dynamics and pathogen-specific biological characteristics. The recent syphilis incidence observed in our cohort (2.3–2.4 per 100 person-years) is broadly comparable to estimates reported in European ART cohorts, such as Croatia (3.3–9.3 per 100 person-years, 2018–2021), and substantially higher than rates reported in some sub-Saharan African ART settings (0.1 per 100 person-years), highlighting considerable cross-setting heterogeneity. In China, national surveillance data demonstrate a marked resurgence of syphilis, with reported incidence increasing from 7.12 per 100,000 in 2004 to 38.37 per 100,000 in 2019, corresponding to an average annual growth rate of 11.9%.^11^ In parallel, pooled estimates indicate that syphilis seroprevalence among PWH in China ranges from 18% to 20%, and exceeds 20% among HIV-positive MSM, far higher than in the general adult population.^22^ These epidemiological patterns underscore the persistence of concentrated transmission within high-prevalence networks in the Western Pacific region.

Several interrelated mechanisms likely contribute to the dominance of syphilis and HPV in the ART era. First, HIV epidemics in the Western Pacific are highly concentrated within densely interconnected MSM sexual networks, enabling efficient parallel transmission of multiple sexually transmitted pathogens. Network clustering, partner concurrency, and high background prevalence amplify reinfection cycles, particularly for bacterial STIs such as syphilis. Second, although ART effectively suppresses HIV replication, it does not confer protection against acquisition or onward transmission of non-HIV STIs. As HIV-related mortality declines and life expectancy increases, prolonged sexual activity within stable transmission networks may sustain high STI incidence despite viral suppression. Third, HPV infection is characterized by viral persistence and impaired clearance in immunocompromised individuals. Even with immune reconstitution following ART initiation, HPV persistence remains common. A recent meta-analysis reported an overall HPV prevalence of approximately 52% among people with HIV in China, with particularly high rates of anal HPV infection among MSM.^19^ Longitudinal data further demonstrate higher persistence and lower clearance of anal HPV in HIV-positive MSM compared with HIV-negative individuals.^23^ Taken together, the convergence of concentrated sexual networks, limited indirect protection from ART against non-HIV pathogens, and the biological persistence of HPV provides a coherent explanation for the continued dominance of syphilis and HPV in the post-ART epidemiological landscape of the Western Pacific region.

HPV remains the second most common STI in our cohort, reflecting both biological vulnerability and policy gaps. In China, HPV vaccine coverage among women was only around 6.2% by 2022, far below the ≥70% coverage achieved in many high-income countries.^24^, ^25^, ^26^ Importantly, male vaccination was not included in the national program until 2025, leaving many MSM, who account for a large proportion of PWH, outside the scope of primary prevention.^27^ This vaccination gap, compounded by persistent viral latency and behavioral risk factors, likely explains the continued HPV burden. Expanding HPV vaccination to PWH, particularly MSM and younger individuals, alongside tailored health education and behavioral interventions, will be essential to reduce morbidity and transmission. The resurgence of bacterial STIs, notably gonorrhea and chlamydia, parallels global surveillance data and may be driven by declining condom use in the era of biomedical HIV prevention, antimicrobial resistance, and gaps in STI screening integration.^28^^,^^29^ The frequent co-occurrence of syphilis and HPV in our cohort reinforces that pathogen-specific approaches are inadequate and integrated prevention is essential.^30^

Marked heterogeneity in STI risk was observed across demographic and clinical subgroups. Younger PWH exhibited the highest likelihood of acquiring STIs, largely attributable to their distinctive behavioral and psychosocial characteristics. Compared with older adults, they are more likely to engage in dynamic, app-mediated sexual networks characterized by multiple or casual partners, greater sensation-seeking, and reduced risk perception.^31^ Men generally had a higher STI risk than women, driven by lower condom use and concurrent partnerships.^32^ This disparity was particularly pronounced among MSM, who demonstrated markedly higher incidence rates of syphilis, HPV, and overall STIs compared with heterosexuals. A global meta-analysis estimated a pooled syphilis prevalence of 10.4% among MSM, with significantly higher rates observed in HIV-positive MSM.^33^ Such differences likely arise from a combination of behavioral and structural factors, including multiple partnerships, recreational drug use, and condomless anal intercourse, as well as stigma, limited healthcare access, and delayed screening, all of which sustain ongoing transmission networks.^34^ Addressing stigma, service fragmentation, and cost barriers is essential for achieving equitable outcomes across the Western Pacific region. By contrast, chlamydia infection was more common among women, reflecting both biological susceptibility—such as increased cervical ectopy and reduced cervical mucus—and greater screening frequency in gynecological settings, whereas low screening coverage among men may lead to underestimation of their contribution to transmissio.^35^ Higher CD4^+^ T cell counts were associated with increased risks of syphilis and HPV, possibly reflecting a “healthy survivor effect,” whereby individuals initiating ART earlier remain sexually active and thus more exposed to infection opportunities. In contrast, HSV-2 incidence was highest among older adults and those with lower CD4^+^ T cell counts, reflecting the combined influence of immunosuppression and viral reactivation, as well as the cumulative nature of lifelong latent infection that leads to an age-related increase in seroprevalence.^36^^,^^37^ Although STI risk appeared to vary across ART regimens, these associations should not be interpreted as evidence of direct pharmacological effects. ART regimen is more likely a surrogate marker of treatment era, baseline patient characteristics, patterns of healthcare engagement, and unmeasured behavioral factors. Therefore, the observed differences in STI incidence across regimens are more plausibly attributable to residual confounding than to causal effects of specific antiretroviral agents.^38^

Beyond first infections, both reinfection and recurrence substantially contributed to the overall STI burden. Among viral infections, HPV and HSV-2 showed the highest recurrence rates, reflecting the challenges of viral persistence and reactivation. These patterns reflect the biology of viral latency—HPV and HSV-2 can persist within epithelial or neuronal reservoirs and periodically reactivate under HIV-related immune dysregulation.^39^^,^^40^ In contrast, bacterial STIs, though curable, showed frequent reinfections—nearly one-quarter of participants experienced syphilis reinfection—indicating ongoing transmission despite treatment. This likely results from poor adherence to antibiotic regimens, absence of lasting immunity, and continued high-risk behaviors after infection, particularly among MSM.^41^ As the CDC notes, no vaccines and few chemoprophylaxis options exist for bacterial STIs; doxycycline post-exposure prophylaxis (DoxyPEP) has recently been proposed for high-risk groups.^41^ Strengthening treatment adherence, expanding prophylactic interventions, and integrating behavioral and biomedical prevention are essential to curb reinfection.

This study underscores the need to move beyond a virus-centric approach to HIV care by systematically integrating STI screening, treatment, and prevention. Firstly, routine HIV services should incorporate regular, non-selective STI screening every 3–6 months for sexually active individuals, particularly MSM, to improve case detection and interrupt transmission. Secondly, expanding HPV vaccination is essential for primary prevention, prioritizing MSM and younger patients, with targeted digital campaigns to enhance uptake. In addition, timely and effective treatment with strengthened antimicrobial stewardship—especially against resistant gonorrhea—and partner notification are vital. Emerging evidence suggests that DoxyPEP can reduce the incidence of certain bacterial STIs among high-risk populations.^42^ Although it was not implemented during our study period in China, DoxyPEP may warrant future evaluation within integrated HIV–STI prevention frameworks in appropriate clinical and public health contexts.^43^ Moreover, digital health tools such as SMS reminders and mHealth platforms can promote adherence and safer behaviors in resource-limited settings. Importantly, HIV programs should not substitute for STI prevention but deliver integrated packages combining behavioral counseling, condom promotion, screening, and vaccination. Finally, strengthening rapid diagnostics, vaccine development, and robust surveillance is critical to support evidence-based policies and curb resistant pathogens. As STI and HIV epidemics continue to converge across the Western Pacific, our findings provide a data-driven foundation to inform regional HIV–STI integration frameworks and advance the WHO 2030 syndemic elimination goals. The integrated prevention framework proposed in this study could inform HIV–STI control strategies across the Western Pacific region, where similar epidemiological patterns are emerging.

This study has several strengths, including a large sample size, multicenter design, and over a decade of longitudinal follow-up, allowing robust assessment of incidence, recurrence, and subgroup heterogeneity. However, several limitations should be noted. Reliance on routine clinical records may have led to underestimation of true incidence due to underdiagnosis or misclassification. The lack of systematically collected behavioral data limited the evaluation of psychosocial and behavioral determinants. Sex was recorded as sex assigned at birth, whereas gender identity was not collected; therefore, gender-related influences on STI risk could not be assessed. Incomplete laboratory testing for some participants may have resulted in underreported recurrence or reinfection events. Finally, the cohort was derived from two urban centers and did not include routinely collected race or ethnicity data, which may limit assessment of broader population heterogeneity and the generalizability of the findings to other settings in the Western Pacific. Despite these limitations, the study provides valuable evidence to inform integrated HIV–STI prevention strategies and offers an empirical foundation for designing future STI management and behavioral interventions.

In conclusion, this long-term multicenter cohort provides the most comprehensive evidence to date on STI epidemiology among PWH in China, revealing a persistent high burden of syphilis and HPV, a sharp rise in bacterial STIs, and notable recurrence of viral infections. Integrating routine opt-out screening, HPV vaccination, and digital health-supported behavioral interventions into HIV programs will be essential to curb reinfection and advance regional health equity.

## Contributors

XX and LQS directly accessed and verified the underlying data. XX and LQS performed the analysis and drafted the original manuscript. YH, FZ, ZXW, YJL, JPH, WJW, YSL, XRL, CYL, DZ, and YYH contributed to data acquisition, interpretation, and manuscript revision. PC, HZL, and JYL supervised the study. XX, LQS, and JYL had full access to all the data in the study. JYL, HZL, and PC were responsible for the decision to submit the manuscript for publication. All authors approved the final version of the manuscript and accept responsibility for the integrity of the work. JYL is the guarantor.

## Data sharing statement

Deidentified participant-level data underlying the results reported in this Article will be available from the corresponding author upon reasonable request from the date of publication. Because the data involve people with HIV, they are not publicly available. Requests for access will be subject to approval by the participating institutions and relevant ethical requirements.

## Declaration of interests

All authors have completed the ICMJE uniform disclosure form and declare no competing interests.
