# Supplementary material for: Incidence, recurrence, and determinants of sexually transmitted infections among people with HIV: a multicenter cohort study in China, 2010–2024, with implications for integrated HIV–STI prevention across the Western Pacific
Source: Lancet Reg Health West Pac. 2026 May 22;70:101879. doi: 10.1016/j.lanwpc.2026.101879 (PMC13221911; doi:10.1016/j.lanwpc.2026.101879)
Supplement: Supplementary Material [file mmc1.docx]

**Supplementary material**

**Supplementary Cohort Profile**3

**Supplementary Table S1. Diagnostic Methods and Case Definitions for STIs**6

**Supplementary Table S2. Diagnostic definitions of STI reinfection and recurrence**8

**Supplementary Table S3. Participant characteristics at ART initiation according to STI diagnosis status.**9

**Supplementary Table S4. Time-stratified subdistribution hazard ratios (sHR) from fully adjusted Fine–Gray models**12

**Supplementary Fig. S1 Flow diagram of participant selection and study inclusion**25

**Supplementary Fig. S2 Time-varying subdistribution hazard ratios from non-proportional Fine–Gray models for Any STIs**..26

**Supplementary Fig. S3 Time-varying subdistribution hazard ratios from non-proportional Fine–Gray models for Syphilis** ..27

**Supplementary Fig. S4 Time-varying subdistribution hazard ratios from non-proportional Fine–Gray models for HPV**..28

**Supplementary Fig. S5 Time-varying subdistribution hazard ratios from non-proportional Fine–Gray models for HSV-2**..29

**Supplementary Fig. S6 Time-varying subdistribution hazard ratios from non-proportional Fine–Gray models for Gonorrhoea**..30

**Supplementary Fig. S7 Time-varying subdistribution hazard ratios from non-proportional Fine–Gray models for Chlamydia**..31

**Supplementary Fig. S8 Annual incidence rates of STIs after excluding participants with only one follow-up visit, 2010–2024..**32

**Supplementary Fig. S9 Aalen–Johansen cumulative incidence curves for any STI, syphilis, HPV, HSV-2, gonorrhea, and chlamydia, stratified by sex**33

**Supplementary Fig. S10 Aalen–Johansen cumulative incidence curves for any STI, syphilis, HPV, HSV-2, gonorrhea, and chlamydia, stratified by CD4 group**34

**Supplementary Cohort Profile**

This study was conducted in two major HIV treatment centers in southern China: the Third People’s Hospital of Shenzhen, the designated hospital for HIV care in Shenzhen, and the Fourth People’s Hospital of Nanning, the largest specialized center for HIV management in Guangxi Province. Together, these hospitals managed more than 34,000 people with HIV (PWH) between 2010 and 2024. Antiretroviral therapy (ART) has been provided free of charge in both sites in accordance with national HIV treatment guidelines. Before ART initiation, all patients undergo comprehensive baseline assessments, including HIV viral load, CD4 and CD8 T-cell counts, screening for common opportunistic infections, routine blood and biochemical tests (liver, renal, and thyroid function), electrocardiography, bone density testing, and screening for co-infections such as cytomegalovirus, hepatitis B virus, hepatitis C virus, syphilis, and human papillomavirus. Detailed medical history and lifestyle information are also recorded. Following ART initiation, patients are scheduled for follow-up visits every three months, which include routine viral load and T-cell subset monitoring, supplemented with additional examinations as clinically indicated. The high reimbursement coverage of local health insurance systems in both Shenzhen and Nanning has contributed to low rates of loss to follow-up, ensuring a large and stable longitudinal cohort with high-quality clinical data for epidemiological research. Missing covariate data (<5% for all variables) were handled using multiple imputation by chained equations. Sensitivity analyses comparing complete-case and imputed models yielded consistent results

The following are some studies we have conducted based on these cohort：

1. Li T, Sun L, He Y, et al. Increasing trends of overweight and obesity in treatment-naive people living with HIV in Shenzhen from 2014 to 2020: an emerging health concern. Front Public Health 2023; 11:1186838.

2. Sun L, He Y, Xu L, et al. Higher Risk of Dyslipidemia With Coformulated Elvitegravir, Cobicistat, Emtricitabine, and Tenofovir Alafenamide than Efavirenz, Lamivudine, and Tenofovir Disoproxil Fumarate Among Antiretroviral-Naive People Living With HIV in China. J Acquir Immune Defic Syndr 2022; 91: S8-S15.

3. Liu J, Hou Y, Sun L, et al. High population-attributable fractions of traditional risk factors for non-AIDS-defining diseases among people living with HIV in China: a cohort study. Emerging Microbes & Infections 2021; 10:416-423.

4. Liu J, Sun L, Hou Y, et al. Barriers to early diagnosis and treatment of persistently high burden of severely immunosuppressed patients with HIV-1: a quantitative and qualitative study. HIV Medicine 2020; 21:708-717.

5. Sun LQ, Liu JY, He Y, et al. Evolution of blood lipids and risk factors of dyslipidemia among people living with human immunodeficiency virus who had received first-line antiretroviral regimens for 3 years in Shenzhen. Chin Med J 2020; 133:2808-2815.

6. Sun L, Luo Y, Jia X, et al. Association between the triglyceride to high-density lipoprotein cholesterol ratio and cardiovascular diseases in people living with human immunodeficiency virus: Evidence from a retrospectively cohort study 2005-2022. Chin Med J (Engl). 2024;137(22):2712-2719.

7. Luo Y, Sun L, He Y, et al. The triglyceride-glucose index trajectories are associated with cardiovascular diseases in people living with HIV: evidence from a prospective cohort study in China, 2005-2022. BMC Public Health. 2025;25(1):465.

8. Li X, Sun L, He Y, et al. HDL-C as a novel predictor of immune reconstitution in people living with HIV: insights from a baseline-to-dynamic change cohort study in China, 2005-2022. Front Immunol. 2025;16:1520615. Published 2025 May 12.

**Supplementary Table S1.** Diagnostic Methods and Case Definitions for STIs

| **Pathogen** | **Diagnostic assay** | **Specimen type** | **Case definition applied in this Study** | **Interpretation criteria** | **Screening strategy** | **Consistency across centers** | **Guideline alignment** |
| --- | --- | --- | --- | --- | --- | --- | --- |
| **Syphilis** | Non-treponemal test (RPR or VDRL) plus treponemal test (TPPA/TPHA/EIA) | Serum | Concurrent reactive non-treponemal and treponemal tests | Interpreted according to CDC-recommended traditional or reverse algorithms | Routine serologic screening during follow-up and symptom-triggered testing | Identical diagnostic algorithms and interpretation thresholds applied at both centers | CDC 2021 STI Treatment Guidelines; WHO 2016 syphilis guideline |
| **Gonorrhea (N. gonorrhoeae)** | Nucleic acid amplification test (NAAT); culture where available | Urogenital, rectal, or pharyngeal specimens | Positive NAAT or culture confirmation | Manufacturer-recommended positivity thresholds; interpreted per CDC guidelines | Risk-based or symptom-triggered testing | Same NAAT platforms and laboratory protocols used across centers | CDC 2021; WHO 2016 gonorrhea guideline |
| **Chlamydia (C. trachomatis)** | Nucleic acid amplification test (NAAT) | Urogenital, rectal, or pharyngeal specimens | Positive NAAT result | Manufacturer-defined positivity criteria | Risk-based or symptom-triggered testing | Uniform assay platforms and interpretation criteria | CDC 2021; WHO 2016 chlamydia guideline |
| **Human Papillomavirus (HPV)** | PCR-based HPV DNA genotyping assays | Cervical, anal, or lesion specimens (as applicable) | Detection of HPV DNA (genotype-specific where available) | Laboratory-defined detection thresholds | Risk-based screening and clinical indication; not universally screened at each visit | Standardized laboratory platforms across centers | CDC 2021 STI Guidelines (HPV section) |
| **Herpes Simplex Virus Type 2 (HSV-2)** | Type-specific glycoprotein G–based serology and/or PCR from lesion specimens | Serum; lesion swabs | Positive type-specific serology and/or PCR detection | Interpreted per manufacturer and CDC-recommended criteria | Symptom-triggered testing | Identical diagnostic algorithms across centers | CDC 2021; WHO 2016 genital herpes guideline |

**Supplementary Table S2.** Diagnostic definitions of STI reinfection and recurrence

| **Pathogen** | **Type of outcome** | **Definition** | **Laboratory/clinical criteria** | **Reference** |
| --- | --- | --- | --- | --- |
| **Syphilis** | Reinfection | ≥4-fold rise in non-treponemal titres (eg, TRUST/RPR) after documented treatment response | Serological testing (non-treponemal + treponemal confirmation) | CDC 2021 STI Guidelines, Syphilis Section |
| **Gonorrhea** | Reinfection | Repeat NAAT or culture positivity ≥30 days after completion of recommended therapy, with interim clinical and exposure assessment used to distinguish reinfection from treatment failure. | NAAT or culture | CDC 2021 STI Guidelines, Gonococcal Infections Section |
| **Chlamydia** | Reinfection | Repeat NAAT positivity after documented completion of recommended therapy, with clinical review to exclude persistent infection. | NAAT testing | CDC 2021 STI Guidelines, Chlamydial Infections Section |
| **HPV** | Recurrence | Detection of the same HPV genotype following prior documented negative testing or clinical resolution. | HPV genotyping (DNA test) | CDC 2021 STI Guidelines (HPV section) |
| **HSV-2** | Recurrence | PCR-confirmed recurrent HSV-2 infection from lesion specimens following prior clinical resolution. | PCR genotyping | CDC 2021 STI Guidelines, Genital Herpes Section |

**Supplementary Table S3**. Participant characteristics at ART initiation according to STI diagnosis status.

| **Characteristics** | **Total** | **No STI** | **Any**  **STI before ART** | **Any**  **STI after ART** | ***P* value** |
| --- | --- | --- | --- | --- | --- |
| **N** | 32,961 | 25,466 | 3331 | 4164 |  |
| **Male, n (%)** | 27,389 (83.1) | 20,395 (80.1) | 3144 (94.4) | 3850 (92.5) | <0.001 |
| **Age, years, n (%)** |  |  |  |  | <0.001 |
| 18-29 | 9974 (30.3) | 6911 (27.1) | 1208 (36.3) | 1855 (44.5) |  |
| 30-39 | 9552 (29.0) | 7106 (27.9) | 1074 (32.2) | 1372 (32.9) |  |
| 40-49 | 5841 (17.7) | 4691 (18.4) | 558 (16.8) | 592 (14.2) |  |
| ≥ 50 | 7594 (23.0) | 6758 (26.5) | 491 (14.7) | 345 (8.3) |  |
| **BMI, kg/m2, n (%)** |  |  |  |  | <0.001 |
| < 18.5 | 6097 (18.5) | 4796 (18.8) | 600 (18.0) | 701 (16.8) |  |
| 18.5–24.9 | 20,486 (62.2) | 15,678 (61.6) | 2083 (62.5) | 2725 (65.4) |  |
| ≥ 25.0 | 6378 (19.4) | 4992 (19.6) | 648 (19.5) | 738 (17.7) |  |
| **Marital status, n (%)** |  |  |  |  | <0.001 |
| Never married | 15,792 (47.9) | 10,833 (42.5) | 2152 (64.6) | 2807 (67.4) |  |
| Married or cohabiting | 13,146 (39.9) | 11,288 (44.3) | 818 (24.6) | 1040 (25.0) |  |
| Divorced, separated, or widowed | 3917 (11.9) | 3254 (12.8) | 353 (10.6) | 310 (7.4) |  |
| Unknown | 106 (0.3) | 91 (0.4) | 8 (0.2) | 7 (0.2) |  |
| **HIV transmission route, n (%)** |  |  |  |  | <0.001 |
| Male-to-male sex contact | 14,378 (43.6) | 9431 (37.0) | 2149 (64.5) | 2798 (67.2) |  |
| Heterosexual contact | 17,128 (52.0) | 14,759 (58.0) | 1100 (33.0) | 1269 (30.5) |  |
| IDU | 753 (2.3) | 682 (2.7) | 36 (1.1) | 35 (0.8) |  |
| Other | 702 (2.1) | 594 (2.3) | 46 (1.4) | 62 (1.5) |  |
| **WBC, 10^9/L** | 5.5 ± 1.9 | 5.4 ± 1.7 | 5.7 ± 1.9 | 5.5 ± 1.6 | <0.001 |
| **Platelet, 10^9/L** | 219.7 ± 73.7 | 217.1 ± 64.5 | 226.4 ± 75.7 | 216.3 ± 60.0 | <0.001 |
| **estimated Glomerular Filtration Rate, mL/min/1.73 m²** | 110.1 ± 18.1 | 111.4 ± 15.5 | 113.6 ± 16.4 | 115.6 ± 13.6 | <0.001 |
| **Alanine Aminotransferase, U/L** | 25.2 ± 15.5 | 23.0 ± 12.1 | 25.0 ± 15.4 | 23.2 ± 11.7 | 0.161 |
| **Aspartate Aminotransferase, U/L** | 25.7 ± 10.3 | 24.4 ± 8.1 | 25.3 ± 10.0 | 23.7 ± 7.5 | <0.001 |
| **Hypercholesterolemia, n (%)** | 2381 (7.2) | 2037 (8.0) | 193 (5.8) | 151 (3.6) | <0.001 |
| **Hypertension, n (%)** | 1060 (3.2) | 886 (3.5) | 117 (3.5) | 57 (1.4) | <0.001 |
| **CKD, n (%)** | 848 (2.6) | 696 (2.7) | 99 (3.0) | 53 (1.3) | <0.001 |
| **Diabetes, n (%)** | 1585 (4.8) | 1358 (5.3) | 136 (4.1) | 91 (2.2) | <0.001 |
| **HBV infection, n (%)** | 3615 (11.0) | 2812 (11.0) | 376 (11.3) | 113 (10.3) | 0.239 |
| **HCV infection, n (%)** | 973 (3.0) | 842 (3.3) | 61 (1.8) | 70 (1.7) | <0.001 |
| **Opportunistic infections, n (%)** | 1222 (3.7) | 871 (3.4) | 188 (5.6) | 163 (3.9) | <0.001 |
| **Time interval, months, n (%)** |  |  |  |  | <0.001 |
| < 1 | 19,404 (58.9) | 15,221 (59.8) | 1883 (56.5) | 2300 (55.2) |  |
| ≥ 1 | 13,557 (41.1) | 10,245 (40.2) | 1448 (43.5) | 1864 (44.8) |  |
| **Initial CD4 count, cells/μL, n (%)** |  |  |  |  | <0.001 |
| < 200 | 13,548 (41.1) | 10,753 (42.2) | 1321 (39.7) | 1474 (35.4) |  |
| 200-349 | 11,005 (33.4) | 8367 (32.9) | 1151 (34.6) | 1487 (35.7) |  |
| ≥ 350 | 8408 (25.5) | 6346 (24.9) | 859 (25.8) | 1203 (28.9) |  |
| **Initial CD8 count, cells/μL, n (%)** |  |  |  |  | <0.001 |
| < 500 | 6480 (19.8) | 5370 (21.3) | 516 (15.6) | 594 (14.4) |  |
| 500-999 | 14,515 (44.4) | 11,170 (44.2) | 1460 (44.2) | 1885 (45.7) |  |
| ≥ 1000 | 11,676 (35.7) | 8705 (34.5) | 1325 (40.1) | 1646 (39.9) |  |
| **Initial HIV RNA, copies/ml, n (%)** |  |  |  |  | <0.001 |
| < 100000 | 15,234 (46.2) | 11,954 (46.9) | 1454 (43.7) | 1826 (43.9) |  |
| ≥ 100000 | 17,727 (53.8) | 13,512 (53.1) | 1877 (56.3) | 2338 (56.1) |  |
| **Types of sexually transmitted diseases, n (%)** |  |  |  |  | <0.001 |
| Syphilis | 5479 (16.6) | 0 (0.0) | 2798 (84.0) | 2681 (64.4) |  |
| HPV | 2588 (7.9) | 0 (0.0) | 820 (24.6) | 1768 (42.5) |  |
| HSV-2 | 146 (0.4) | 0 (0.0) | 68 (2.0) | 78 (1.9) |  |
| Gonorrhea | 82 (0.2) | 0 (0.0) | 23 (0.7) | 59 (1.4) |  |
| Chlamydia | 102 (0.3) | 0 (0.0) | 31 (0.9) | 71 (1.7) |  |
| **ART treatment regimen, n (%)** |  |  |  |  | <0.001 |
| 2NRTIs+EFV | 22,654 (68.7) | 17,443 (68.5) | 2086 (62.6) | 3125 (75.0) |  |
| 2NRTIs+LPV/r | 2323 (7.0) | 1903 (7.5) | 210 (6.3) | 210 (5.0) |  |
| BIC/FTC/TAF | 2080 (6.3) | 1518 (6.0) | 383 (11.5) | 179 (4.3) |  |
| 2NRTIs+NVP | 1866 (5.7) | 1582 (6.2) | 49 (1.5) | 235 (5.6) |  |
| DTG-containing | 1715 (5.2) | 1279 (5.0) | 283 (8.5) | 153 (3.7) |  |
| EVG/c/FTC/TAF | 775 (2.4) | 523 (2.1) | 127 (3.8) | 125 (3.0) |  |
| 3TC+LPV/r | 719 (2.2) | 601 (2.4) | 77 (2.3) | 41 (1.0) |  |
| 2NRTIs+ANV/DOR | 287 (0.9) | 235 (0.9) | 31 (0.9) | 21 (0.5) |  |
| Others | 542 (1.6) | 382 (1.5) | 85 (2.6) | 75 (1.8) |  |

Continuous variables are presented as mean ± standard deviation (SD) and compared using one-way analysis of variance (ANOVA). Categorical variables are presented as frequency (%) and compared using Pearson’s χ² test. Time interval refers to the duration between HIV diagnosis and ART initiation.

STI, sexually transmitted infection; ART, antiretroviral therapy; BMI, body-mass index; HIV, human immunodeficiency virus; IDU, injection drug use; WBC, white blood cells; CKD, **chronic kidney disease;** HBV, hepatitis B virus; HCV, hepatitis C virus; HPV, human papillomavirus; HSV-2, herpes simplex virus type 2; NRTIs, nucleoside reverse transcriptase inhibitors; ANV, ainuovirine; NVP, nevirapine; LPV/r, lopinavir/ritonavir; EFV, efavirenz; DOR, doravirine; BIC, bictegravir; FTC, emtricitabine; TAF, tenofovir alafenamide; EVG, elvitegravir; DTG, dolutegravir.

**Supplementary Table S4.** Time-stratified subdistribution hazard ratios (sHR) from fully adjusted Fine–Gray models

| **Characteristics** | **0–1 year** | |  | **1–3 years** | |  | **3–τ years** | |
| --- | --- | --- | --- | --- | --- | --- | --- | --- |
|  | **sHR (95% CI)** | **P** |  | **sHR (95% CI)** | **P** |  | **sHR (95% CI)** | ***P*** |
| **Any STI (τ=9)** |  |  |  |  |  |  |  |  |
| Marital status |  |  |  |  |  |  |  |  |
| Married or cohabiting | 1.00 (reference) |  |  | 1.00 (reference) |  |  | 1.00 (reference) |  |
| Never married | 1.21 (1.02–1.43) | 0.025 |  | 1.47 (1.23–1.76) | <0.001 |  | 1.66 (1.43–1.92) | <0.001 |
| Divorced/widowed | 1.02 (0.82–1.28) | 0.840 |  | 1.13 (0.86–1.48) | 0.373 |  | 1.33 (1.08–1.64) | 0.007 |
| Unknown | 0.76 (0.19–3.06) | 0.703 |  | 1.06 (0.27–4.20) | 0.933 |  | 1.55 (0.50–4.82) | 0.450 |
| BMI, kg/m² |  |  |  |  |  |  |  |  |
| 18.5–24.9 | 1.00 (reference) |  |  | 1.00 (reference) |  |  | 1.00 (reference) |  |
| <18.5 | 0.98 (0.85–1.13) | 0.805 |  | 1.11 (0.94–1.31) | 0.224 |  | 1.07 (0.93–1.24) | 0.333 |
| ≥25.0 | 0.94 (0.78–1.12) | 0.479 |  | 0.97 (0.78–1.20) | 0.778 |  | 1.06 (0.88–1.28) | 0.522 |
| WBC |  |  |  |  |  |  |  |  |
| Normal | 1.00 (reference) |  |  | 1.00 (reference) |  |  | 1.00 (reference) |  |
| Abnormal | 1.02 (0.89–1.18) | 0.749 |  | 1.01 (0.86–1.20) | 0.86 |  | 0.96 (0.83–1.10) | 0.534 |
| Platelet |  |  |  |  |  |  |  |  |
| Normal | 1.00 (reference) |  |  | 1.00 (reference) |  |  | 1.00 (reference) |  |
| Abnormal | 0.98 (0.84–1.15) | 0.822 |  | 1.04 (0.87–1.24) | 0.656 |  | 0.96 (0.82–1.12) | 0.582 |
| eGFR |  |  |  |  |  |  |  |  |
| Normal | 1.00 (reference) |  |  | 1.00 (reference) |  |  | 1.00 (reference) |  |
| Abnormal | 0.73 (0.58–0.92) | 0.009 |  | 0.70 (0.53–0.93) | 0.014 |  | 0.97 (0.78–1.20) | 0.766 |
| ALT |  |  |  |  |  |  |  |  |
| Normal | 1.00 (reference) |  |  | 1.00 (reference) |  |  | 1.00 (reference) |  |
| Abnormal | 0.95 (0.80–1.12) | 0.554 |  | 0.85 (0.71–1.04) | 0.109 |  | 0.97 (0.83–1.14) | 0.718 |
| AST |  |  |  |  |  |  |  |  |
| Normal | 1.00 (reference) |  |  | 1.00 (reference) |  |  | 1.00 (reference) |  |
| Abnormal | 0.92 (0.75–1.14) | 0.449 |  | 1.07 (0.85–1.35) | 0.571 |  | 1.03 (0.84–1.25) | 0.802 |
| Initial CD8 count (cells/μL) |  |  |  |  |  |  |  |  |
| 500–999 | 1.00 (reference) |  |  | 1.00 (reference) |  |  | 1.00 (reference) |  |
| <500 | 0.85 (0.72–1.01) | 0.07 |  | 0.99 (0.81–1.19) | 0.884 |  | 0.86 (0.72–1.01) | 0.07 |
| ≥1000 | 1.05 (0.93–1.18) | 0.439 |  | 1.12 (0.98–1.28) | 0.087 |  | 0.99 (0.89–1.11) | 0.907 |
| Initial HIV RNA (copies/mL) |  |  |  |  |  |  |  |  |
| <100,000 | 1.00 (reference) |  |  | 1.00 (reference) |  |  | 1.00 (reference) |  |
| ≥100,000 | 1.17 (1.05–1.30) | 0.005 |  | 1.14 (1.01–1.29) | 0.032 |  | 1.07 (0.97–1.19) | 0.193 |
| Time interval, months |  |  |  |  |  |  |  |  |
| <1 | 1.00 (reference) |  |  | 1.00 (reference) |  |  | 1.00 (reference) |  |
| ≥1 | 0.83 (0.75–0.93) | 0.001 |  | 0.89 (0.79–1.00) | 0.052 |  | 0.90 (0.81–1.00) | 0.049 |
| Diabetes |  |  |  |  |  |  |  |  |
| No | 1.00 (reference) |  |  | 1.00 (reference) |  |  | 1.00 (reference) |  |
| Yes | 0.62 (0.40–0.96) | 0.034 |  | 0.61 (0.39–0.98) | 0.04 |  | 0.82 (0.57–1.17) | 0.276 |
| CKD |  |  |  |  |  |  |  |  |
| No | 1.00 (reference) |  |  | 1.00 (reference) |  |  | 1.00 (reference) |  |
| Yes | 1.38 (0.90–2.11) | 0.141 |  | 1.26 (0.72–2.20) | 0.426 |  | 1.15 (0.65–2.04) | 0.633 |
| HBV infection |  |  |  |  |  |  |  |  |
| No | 1.00 (reference) |  |  | 1.00 (reference) |  |  | 1.00 (reference) |  |
| Yes | 1.01 (0.84–1.21) | 0.928 |  | 1.09 (0.90–1.31) | 0.385 |  | 0.84 (0.71–1.01) | 0.057 |
| HCV infection |  |  |  |  |  |  |  |  |
| No | 1.00 (reference) |  |  | 1.00 (reference) |  |  | 1.00 (reference) |  |
| Yes | 1.00 (0.67–1.49) | 0.987 |  | 0.61 (0.36–1.04) | 0.068 |  | 0.75 (0.52–1.10) | 0.137 |
| Hypercholesterolemia |  |  |  |  |  |  |  |  |
| No | 1.00 (reference) |  |  | 1.00 (reference) |  |  | 1.00 (reference) |  |
| Yes | 0.86 (0.61–1.20) | 0.377 |  | 1.30 (0.92–1.84) | 0.139 |  | 1.08 (0.80–1.45) | 0.636 |
| Hypertension |  |  |  |  |  |  |  |  |
| No | 1.00 (reference) |  |  | 1.00 (reference) |  |  | 1.00 (reference) |  |
| Yes | 1.22 (0.83–1.80) | 0.311 |  | 0.96 (0.55–1.70) | 0.898 |  | 1.02 (0.60–1.74) | 0.94 |
| Opportunistic infections |  |  |  |  |  |  |  |  |
| No | 1.00 (reference) |  |  | 1.00 (reference) |  |  | 1.00 (reference) |  |
| Yes | 0.94 (0.69–1.29) | 0.71 |  | 1.04 (0.74–1.45) | 0.835 |  | 1.16 (0.90–1.50) | 0.253 |
| ART regimen (collapsed) |  |  |  |  |  |  |  |  |
| NNRTI-based | 1.00 (reference) |  |  | 1.00 (reference) |  |  | 1.00 (reference) |  |
| INSTI-based | 1.48 (1.28–1.71) | <0.001 |  | 1.57 (1.31–1.87) | <0.001 |  | 1.53 (1.14–2.07) | 0.005 |
| PI-based | 1.21 (0.97–1.51) | 0.085 |  | 1.09 (0.83–1.43) | 0.538 |  | 1.07 (0.85–1.34) | 0.564 |
| **Syphilis (τ=9)** | | | | | | | | |
| Marital status |  |  |  |  |  |  |  |  |
| Married or cohabiting | 1.00 (reference) |  |  | 1.00 (reference) |  |  | 1.00 (reference) |  |
| Never married | 1.50 (1.17–1.92) | 0.001 |  | 1.30 (1.04–1.64) | 0.022 |  | 1.55 (1.31–1.84) | <0.001 |
| Divorced/widowed | 1.11 (0.80–1.54) | 0.523 |  | 1.28 (0.93–1.77) | 0.133 |  | 1.32 (1.03–1.67) | 0.025 |
| Unknown | – | – |  | 0.85 (0.12–5.92) | 0.873 |  | 2.24 (0.71–7.08) | 0.171 |
| BMI, kg/m² |  |  |  |  |  |  |  |  |
| 18.5–24.9 | 1.00 (reference) |  |  | 1.00 (reference) |  |  | 1.00 (reference) |  |
| <18.5 | 1.03 (0.84–1.28) | 0.756 |  | 1.22 (0.98–1.51) | 0.074 |  | 1.08 (0.92–1.27) | 0.356 |
| ≥25.0 | 1.01 (0.78–1.32) | 0.931 |  | 1.03 (0.79–1.36) | 0.817 |  | 1.09 (0.89–1.34) | 0.4 |
| WBC |  |  |  |  |  |  |  |  |
| Normal | 1.00 (reference) |  |  | 1.00 (reference) |  |  | 1.00 (reference) |  |
| Abnormal | 0.96 (0.77–1.19) | 0.694 |  | 0.97 (0.78–1.20) | 0.75 |  | 1.05 (0.89–1.23) | 0.549 |
| Platelet |  |  |  |  |  |  |  |  |
| Normal | 1.00 (reference) |  |  | 1.00 (reference) |  |  | 1.00 (reference) |  |
| Abnormal | 0.80 (0.62–1.03) | 0.084 |  | 1.05 (0.84–1.31) | 0.659 |  | 0.97 (0.82–1.15) | 0.727 |
| eGFR |  |  |  |  |  |  |  |  |
| Normal | 1.00 (reference) |  |  | 1.00 (reference) |  |  | 1.00 (reference) |  |
| Abnormal | 0.77 (0.55–1.09) | 0.136 |  | 0.78 (0.54–1.12) | 0.18 |  | 0.96 (0.74–1.24) | 0.765 |
| ALT |  |  |  |  |  |  |  |  |
| Normal | 1.00 (reference) |  |  | 1.00 (reference) |  |  | 1.00 (reference) |  |
| Abnormal | 0.97 (0.76–1.23) | 0.791 |  | 0.76 (0.60–0.96) | 0.02 |  | 0.89 (0.75–1.07) | 0.23 |
| AST |  |  |  |  |  |  |  |  |
| Normal | 1.00 (reference) |  |  | 1.00 (reference) |  |  | 1.00 (reference) |  |
| Abnormal | 1.06 (0.79–1.41) | 0.701 |  | 1.03 (0.77–1.37) | 0.863 |  | 1.11 (0.89–1.39) | 0.343 |
| Initial CD8 count (cells/μL) |  |  |  |  |  |  |  |  |
| 500–999 | 1.00 (reference) |  |  | 1.00 (reference) |  |  | 1.00 (reference) |  |
| <500 | 0.90 (0.70–1.15) | 0.391 |  | 1.02 (0.80–1.30) | 0.885 |  | 0.80 (0.66–0.98) | 0.032 |
| ≥1000 | 0.92 (0.77–1.09) | 0.313 |  | 1.14 (0.97–1.34) | 0.105 |  | 1.09 (0.96–1.23) | 0.194 |
| Initial HIV RNA (copies/mL) |  |  |  |  |  |  |  |  |
| <100,000 | 1.00 (reference) |  |  | 1.00 (reference) |  |  | 1.00 (reference) |  |
| ≥100,000 | 1.25 (1.07–1.46) | 0.005 |  | 1.11 (0.95–1.28) | 0.181 |  | 1.08 (0.96–1.21) | 0.205 |
| Time interval, months |  |  |  |  |  |  |  |  |
| <1 | 1.00 (reference) |  |  | 1.00 (reference) |  |  | 1.00 (reference) |  |
| ≥1 | 0.87 (0.75–1.02) | 0.093 |  | 1.01 (0.87–1.18) | 0.846 |  | 0.92 (0.82–1.03) | 0.154 |
| Diabetes |  |  |  |  |  |  |  |  |
| No | 1.00 (reference) |  |  | 1.00 (reference) |  |  | 1.00 (reference) |  |
| Yes | 0.69 (0.37–1.28) | 0.237 |  | 0.67 (0.38–1.17) | 0.158 |  | 1.03 (0.68–1.56) | 0.899 |
| CKD |  |  |  |  |  |  |  |  |
| No | 1.00 (reference) |  |  | 1.00 (reference) |  |  | 1.00 (reference) |  |
| Yes | 1.31 (0.71–2.43) | 0.385 |  | 0.92 (0.39–2.16) | 0.853 |  | 1.38 (0.73–2.59) | 0.316 |
| HBV infection |  |  |  |  |  |  |  |  |
| No | 1.00 (reference) |  |  | 1.00 (reference) |  |  | 1.00 (reference) |  |
| Yes | 1.07 (0.83–1.37) | 0.598 |  | 1.13 (0.89–1.42) | 0.318 |  | 0.82 (0.68–1.00) | 0.054 |
| HCV infection |  |  |  |  |  |  |  |  |
| No | 1.00 (reference) |  |  | 1.00 (reference) |  |  | 1.00 (reference) |  |
| Yes | 1.31 (0.79–2.19) | 0.3 |  | 0.68 (0.35–1.33) | 0.258 |  | 0.70 (0.45–1.10) | 0.121 |
| Hypercholesterolemia |  |  |  |  |  |  |  |  |
| No | 1.00 (reference) |  |  | 1.00 (reference) |  |  | 1.00 (reference) |  |
| Yes | 0.90 (0.55–1.46) | 0.661 |  | 1.64 (1.07–2.49) | 0.022 |  | 0.89 (0.61–1.30) | 0.561 |
| Hypertension |  |  |  |  |  |  |  |  |
| No | 1.00 (reference) |  |  | 1.00 (reference) |  |  | 1.00 (reference) |  |
| Yes | 1.18 (0.68–2.06) | 0.557 |  | 1.04 (0.52–2.06) | 0.913 |  | 1.05 (0.58–1.91) | 0.875 |
| Opportunistic infections |  |  |  |  |  |  |  |  |
| No | 1.00 (reference) |  |  | 1.00 (reference) |  |  | 1.00 (reference) |  |
| Yes | 1.16 (0.75–1.79) | 0.499 |  | 1.12 (0.74–1.70) | 0.581 |  | 1.14 (0.85–1.53) | 0.374 |
| ART regimen (collapsed) |  |  |  |  |  |  |  |  |
| NNRTI-based | 1.00 (reference) |  |  | 1.00 (reference) |  |  | 1.00 (reference) |  |
| INSTI-based | 1.49 (1.21–1.82) | <0.001 |  | 1.79 (1.45–2.21) | <0.001 |  | 1.48 (1.06–2.08) | 0.022 |
| PI-based | 1.11 (0.80–1.55) | 0.534 |  | 0.99 (0.69–1.43) | 0.971 |  | 0.91 (0.69–1.20) | 0.515 |
| **HPV (τ=7)** | | | | | | | | |
| Marital status |  |  |  |  |  |  |  |  |
| Married or cohabiting | 1.00 (reference) |  |  | 1.00 (reference) |  |  | 1.00 (reference) |  |
| Never married | 1.14 (0.93–1.40) | 0.214 |  | 1.63 (1.25–2.13) | <0.001 |  | 1.92 (1.44–2.55) | <0.001 |
| Divorced/widowed | 0.91 (0.67–1.22) | 0.513 |  | 0.87 (0.57–1.34) | 0.534 |  | 1.36 (0.91–2.05) | 0.137 |
| Unknown | 1.36 (0.34–5.44) | 0.668 |  | 1.14 (0.16–8.28) | 0.895 |  | 1.64 (0.22–12.06) | 0.629 |
| BMI, kg/m² |  |  |  |  |  |  |  |  |
| 18.5–24.9 | 1.00 (reference) |  |  | 1.00 (reference) |  |  | 1.00 (reference) |  |
| <18.5 | 0.96 (0.81–1.15) | 0.682 |  | 0.94 (0.76–1.17) | 0.597 |  | 1.07 (0.82–1.39) | 0.619 |
| ≥25.0 | 1.00 (0.80–1.26) | 0.972 |  | 0.91 (0.68–1.22) | 0.527 |  | 0.90 (0.63–1.28) | 0.557 |
| WBC |  |  |  |  |  |  |  |  |
| Normal | 1.00 (reference) |  |  | 1.00 (reference) |  |  | 1.00 (reference) |  |
| Abnormal | 1.00 (0.84–1.20) | 0.979 |  | 1.10 (0.88–1.37) | 0.393 |  | 0.96 (0.74–1.24) | 0.736 |
| Platelet |  |  |  |  |  |  |  |  |
| Normal | 1.00 (reference) |  |  | 1.00 (reference) |  |  | 1.00 (reference) |  |
| Abnormal | 1.04 (0.85–1.27) | 0.711 |  | 1.13 (0.89–1.44) | 0.312 |  | 0.97 (0.73–1.29) | 0.828 |
| eGFR |  |  |  |  |  |  |  |  |
| Normal | 1.00 (reference) |  |  | 1.00 (reference) |  |  | 1.00 (reference) |  |
| Abnormal | 0.68 (0.50–0.94) | 0.019 |  | 0.61 (0.40–0.92) | 0.018 |  | 1.09 (0.72–1.63) | 0.692 |
| ALT |  |  |  |  |  |  |  |  |
| Normal | 1.00 (reference) |  |  | 1.00 (reference) |  |  | 1.00 (reference) |  |
| Abnormal | 0.86 (0.69–1.07) | 0.169 |  | 1.00 (0.77–1.31) | 0.971 |  | 1.04 (0.78–1.40) | 0.79 |
| AST |  |  |  |  |  |  |  |  |
| Normal | 1.00 (reference) |  |  | 1.00 (reference) |  |  | 1.00 (reference) |  |
| Abnormal | 0.82 (0.62–1.08) | 0.151 |  | 1.01 (0.73–1.40) | 0.932 |  | 1.09 (0.76–1.56) | 0.628 |
| Initial CD8 count (cells/μL) |  |  |  |  |  |  |  |  |
| 500–999 | 1.00 (reference) |  |  | 1.00 (reference) |  |  | 1.00 (reference) |  |
| <500 | 0.80 (0.64–1.00) | 0.051 |  | 0.98 (0.75–1.28) | 0.899 |  | 1.00 (0.74–1.35) | 0.991 |
| ≥1000 | 1.13 (0.98–1.31) | 0.094 |  | 1.17 (0.96–1.41) | 0.115 |  | 0.95 (0.76–1.18) | 0.645 |
| Initial HIV RNA (copies/mL) |  |  |  |  |  |  |  |  |
| <100,000 | 1.00 (reference) |  |  | 1.00 (reference) |  |  | 1.00 (reference) |  |
| ≥100,000 | 1.16 (1.01–1.33) | 0.033 |  | 1.24 (1.04–1.48) | 0.016 |  | 0.96 (0.78–1.17) | 0.658 |
| Time interval, months |  |  |  |  |  |  |  |  |
| <1 | 1.00 (reference) |  |  | 1.00 (reference) |  |  | 1.00 (reference) |  |
| ≥1 | 0.82 (0.71–0.94) | 0.005 |  | 0.75 (0.63–0.89) | 0.001 |  | 0.94 (0.77–1.15) | 0.565 |
| Diabetes |  |  |  |  |  |  |  |  |
| No | 1.00 (reference) |  |  | 1.00 (reference) |  |  | 1.00 (reference) |  |
| Yes | 0.62 (0.34–1.13) | 0.12 |  | 0.44 (0.18–1.05) | 0.063 |  | 0.62 (0.31–1.25) | 0.181 |
| CKD |  |  |  |  |  |  |  |  |
| No | 1.00 (reference) |  |  | 1.00 (reference) |  |  | 1.00 (reference) |  |
| Yes | 1.49 (0.88–2.52) | 0.133 |  | 1.61 (0.83–3.10) | 0.157 |  | 0.46 (0.12–1.79) | 0.26 |
| HBV infection |  |  |  |  |  |  |  |  |
| No | 1.00 (reference) |  |  | 1.00 (reference) |  |  | 1.00 (reference) |  |
| Yes | 1.01 (0.80–1.26) | 0.948 |  | 0.98 (0.75–1.30) | 0.905 |  | 1.10 (0.82–1.49) | 0.513 |
| HCV infection |  |  |  |  |  |  |  |  |
| No | 1.00 (reference) |  |  | 1.00 (reference) |  |  | 1.00 (reference) |  |
| Yes | 0.74 (0.40–1.35) | 0.318 |  | 0.63 (0.29–1.36) | 0.24 |  | 0.45 (0.19–1.11) | 0.084 |
| Hypercholesterolemia |  |  |  |  |  |  |  |  |
| No | 1.00 (reference) |  |  | 1.00 (reference) |  |  | 1.00 (reference) |  |
| Yes | 0.77 (0.48–1.25) | 0.296 |  | 0.83 (0.45–1.52) | 0.546 |  | 1.32 (0.79–2.23) | 0.291 |
| Hypertension |  |  |  |  |  |  |  |  |
| No | 1.00 (reference) |  |  | 1.00 (reference) |  |  | 1.00 (reference) |  |
| Yes | 1.40 (0.87–2.24) | 0.164 |  | 0.95 (0.42–2.14) | 0.893 |  | 1.03 (0.38–2.73) | 0.96 |
| Opportunistic infections |  |  |  |  |  |  |  |  |
| No | 1.00 (reference) |  |  | 1.00 (reference) |  |  | 1.00 (reference) |  |
| Yes | 1.24 (0.89–1.73) | 0.203 |  | 0.89 (0.55–1.41) | 0.61 |  | 0.95 (0.57–1.57) | 0.831 |
| ART regimen (collapsed) |  |  |  |  |  |  |  |  |
| NNRTI-based | 1.00 (reference) |  |  | 1.00 (reference) |  |  | 1.00 (reference) |  |
| INSTI-based | 1.57 (1.32–1.88) | <0.001 |  | 1.47 (1.15–1.88) | 0.002 |  | 2.24 (1.47–3.41) | <0.001 |
| PI-based | 1.33 (1.02–1.73) | 0.037 |  | 1.38 (0.99–1.94) | 0.058 |  | 1.36 (0.94–1.98) | 0.102 |
| **HSV-2 (τ=8.5)** | | | | | | | | |
| Marital status |  |  |  |  |  |  |  |  |
| Married or cohabiting | 1.00 (reference) |  |  | 1.00 (reference) |  |  | 1.00 (reference) |  |
| Never married | 0.39 (0.09–1.66) | 0.203 |  | 1.71 (0.58–5.09) | 0.333 |  | 2.47 (0.97–6.28) | 0.058 |
| Divorced/widowed | 2.23 (1.04–4.78) | 0.039 |  | 1.05 (0.20–5.52) | 0.95 |  | 1.29 (0.33–5.05) | 0.713 |
| Unknown | - | - |  | 17.57 (1.57–196.31) | 0.02 |  | - | - |
| BMI, kg/m² |  |  |  |  |  |  |  |  |
| 18.5–24.9 | 1.00 (reference) |  |  | 1.00 (reference) |  |  | 1.00 (reference) |  |
| <18.5 | 1.42 (0.57–3.52) | 0.455 |  | 0.67 (0.28–1.59) | 0.365 |  | 1.84 (0.48–7.01) | 0.373 |
| ≥25.0 | 1.40 (0.46–4.27) | 0.557 |  | - | - |  | 1.46 (0.31–6.90) | 0.636 |
| WBC |  |  |  |  |  |  |  |  |
| Normal | 1.00 (reference) |  |  | 1.00 (reference) |  |  | 1.00 (reference) |  |
| Abnormal | 0.83 (0.40–1.73) | 0.624 |  | 0.48 (0.14–1.62) | 0.239 |  | 0.33 (0.10–1.12) | 0.076 |
| Platelet |  |  |  |  |  |  |  |  |
| Normal | 1.00 (reference) |  |  | 1.00 (reference) |  |  | 1.00 (reference) |  |
| Abnormal | 1.79 (0.88–3.66) | 0.108 |  | 0.18 (0.02–1.51) | 0.113 |  | 1.17 (0.41–3.30) | 0.766 |
| eGFR |  |  |  |  |  |  |  |  |
| Normal | 1.00 (reference) |  |  | 1.00 (reference) |  |  | 1.00 (reference) |  |
| Abnormal | 1.09 (0.51–2.36) | 0.819 |  | 1.25 (0.30–5.26) | 0.758 |  | 2.76 (0.87–8.76) | 0.084 |
| ALT |  |  |  |  |  |  |  |  |
| Normal | 1.00 (reference) |  |  | 1.00 (reference) |  |  | 1.00 (reference) |  |
| Abnormal | 0.79 (0.30–2.05) | 0.629 |  | 0.26 (0.07–0.95) | 0.042 |  | 0.40 (0.08–1.96) | 0.261 |
| AST |  |  |  |  |  |  |  |  |
| Normal | 1.00 (reference) |  |  | 1.00 (reference) |  |  | 1.00 (reference) |  |
| Abnormal | 1.36 (0.54–3.40) | 0.517 |  | 4.89 (1.47–16.24) | 0.009 |  | 1.13 (0.20–6.46) | 0.889 |
| Initial CD8 count (cells/μL) |  |  |  |  |  |  |  |  |
| 500–999 (ref) |  |  |  |  |  |  |  |  |
| <500 | 0.64 (0.28–1.44) | 0.281 |  | 1.17 (0.36–3.79) | 0.79 |  | 0.42 (0.09–1.88) | 0.257 |
| ≥1000 | 0.51 (0.19–1.33) | 0.165 |  | 1.43 (0.59–3.50) | 0.429 |  | 1.07 (0.49–2.32) | 0.871 |
| Initial HIV RNA (copies/mL) |  |  |  |  |  |  |  |  |
| <100,000 | 1.00 (reference) |  |  | 1.00 (reference) |  |  | 1.00 (reference) |  |
| ≥100,000 | 0.71 (0.37–1.39) | 0.319 |  | 0.67 (0.30–1.49) | 0.323 |  | 0.81 (0.36–1.79) | 0.596 |
| Time interval, months |  |  |  |  |  |  |  |  |
| <1 | 1.00 (reference) |  |  | 1.00 (reference) |  |  | 1.00 (reference) |  |
| ≥1 | 1.53 (0.76–3.09) | 0.232 |  | 0.88 (0.38–2.04) | 0.771 |  | 0.45 (0.20–1.02) | 0.056 |
| Diabetes |  |  |  |  |  |  |  |  |
| No | 1.00 (reference) |  |  | 1.00 (reference) |  |  | 1.00 (reference) |  |
| Yes | 0.25 (0.03–1.89) | 0.178 |  | 3.57 (1.38–9.24) | 0.009 |  | 0.90 (0.05–16.36) | 0.942 |
| CKD |  |  |  |  |  |  |  |  |
| No | 1.00 (reference) |  |  | 1.00 (reference) |  |  | 1.00 (reference) |  |
| Yes | 0.46 (0.07–3.02) | 0.418 |  | 5.92 (0.69–50.48) | 0.104 |  | - | - |
| HBV infection |  |  |  |  |  |  |  |  |
| No | 1.00 (reference) |  |  | 1.00 (reference) |  |  | 1.00 (reference) |  |
| Yes | 0.61 (0.18–2.01) | 0.413 |  | 1.54 (0.51–4.66) | 0.444 |  | 0.63 (0.15–2.64) | 0.531 |
| HCV infection |  |  |  |  |  |  |  |  |
| No | 1.00 (reference) |  |  | 1.00 (reference) |  |  | 1.00 (reference) |  |
| Yes | 0.59 (0.08–4.55) | 0.613 |  | 1.01 (0.11–8.82) | 0.996 |  | - | - |
| Hypercholesterolemia |  |  |  |  |  |  |  |  |
| No | 1.00 (reference) |  |  | 1.00 (reference) |  |  | 1.00 (reference) |  |
| Yes | 2.05 (0.74–5.67) | 0.168 |  | 0.81 (0.32–2.05) | 0.652 |  | 0.57 (0.04–8.69) | 0.686 |
| Hypertension |  |  |  |  |  |  |  |  |
| No | 1.00 (reference) |  |  | 1.00 (reference) |  |  | 1.00 (reference) |  |
| Yes | 0.48 (0.07–3.15) | 0.444 |  | - | - |  | - | - |
| Opportunistic infections |  |  |  |  |  |  |  |  |
| No | 1.00 (reference) |  |  | 1.00 (reference) |  |  | 1.00 (reference) |  |
| Yes | 1.62 (0.49–5.34) | 0.43 |  | 0.76 (0.10–5.92) | 0.79 |  | 1.60 (0.38–6.78) | 0.52 |
| ART regimen (collapsed) |  |  |  |  |  |  |  |  |
| NNRTI-based | 1.00 (reference) |  |  | 1.00 (reference) |  |  | 1.00 (reference) |  |
| INSTI-based | 1.69 (0.61–4.68) | 0.313 |  | 0.89 (0.20–3.97) | 0.88 |  | - | - |
| PI-based | 2.70 (1.23–5.90) | 0.013 |  | 1.41 (0.37–5.31) | 0.616 |  | 3.19 (1.28–7.94) | 0.013 |
| **Gonorrhea (τ=8)** | | | | | | | | |
| Marital status |  |  |  |  |  |  |  |  |
| Married or cohabiting | 1.00 (reference) |  |  | 1.00 (reference) |  |  | 1.00 (reference) |  |
| Never married | 2.17 (0.33–14.51) | 0.423 |  | 0.66 (0.18–2.43) | 0.531 |  | 1.33 (0.39–4.49) | 0.65 |
| Divorced/widowed | - | - |  | 2.34 (0.45–12.29) | 0.315 |  | 3.95 (0.86–18.20) | 0.078 |
| Unknown | - | - |  | - | - |  | - | - |
| BMI, kg/m² |  |  |  |  |  |  |  |  |
| 18.5–24.9 | 1.00 (reference) |  |  | 1.00 (reference) |  |  | 1.00 (reference) |  |
| <18.5 | 0.34 (0.13–0.90) | 0.029 |  | 1.04 (0.29–3.71) | 0.949 |  | 0.62 (0.24–1.56) | 0.308 |
| ≥25.0 | 0.51 (0.13–2.05) | 0.341 |  | 0.50 (0.07–3.35) | 0.475 |  | 1.62 (0.51–5.21) | 0.415 |
| WBC |  |  |  |  |  |  |  |  |
| Normal | 1.00 (reference) |  |  | 1.00 (reference) |  |  | 1.00 (reference) |  |
| Abnormal | 1.90 (0.55–6.55) | 0.309 |  | 0.77 (0.22–2.76) | 0.691 |  | 1.85 (0.76–4.54) | 0.176 |
| Platelet |  |  |  |  |  |  |  |  |
| Normal | 1.00 (reference) |  |  | 1.00 (reference) |  |  | 1.00 (reference) |  |
| Abnormal | 0.39 (0.04–3.60) | 0.407 |  | 0.49 (0.06–4.09) | 0.511 |  | 0.65 (0.18–2.32) | 0.508 |
| eGFR |  |  |  |  |  |  |  |  |
| Normal | 1.00 (reference) |  |  | 1.00 (reference) |  |  | 1.00 (reference) |  |
| Abnormal | - | - |  | 0.56 (0.02–17.20) | 0.743 |  | 3.96 (1.28–12.27) | 0.017 |
| ALT |  |  |  |  |  |  |  |  |
| Normal | 1.00 (reference) |  |  | 1.00 (reference) |  |  | 1.00 (reference) |  |
| Abnormal | 1.00 (0.28–3.63) | 0.997 |  | 0.42 (0.15–1.22) | 0.113 |  | 0.39 (0.11–1.41) | 0.152 |
| AST |  |  |  |  |  |  |  |  |
| Normal | 1.00 (reference) |  |  | 1.00 (reference) |  |  | 1.00 (reference) |  |
| Abnormal | 0.37 (0.05–2.81) | 0.334 |  | 2.45 (1.02–5.91) | 0.046 |  | 1.64 (0.41–6.60) | 0.488 |
| Initial CD8 count (cells/μL) |  |  |  |  |  |  |  |  |
| 500–999 | 1.00 (reference) |  |  | 1.00 (reference) |  |  | 1.00 (reference) |  |
| <500 | 5.47 (1.21–24.86) | 0.028 |  | - | - |  | 2.47 (0.84–7.27) | 0.101 |
| ≥1000 | 2.47 (0.69–8.78) | 0.162 |  | 1.02 (0.37–2.81) | 0.968 |  | 2.01 (0.85–4.74) | 0.112 |
| Initial HIV RNA (copies/mL) |  |  |  |  |  |  |  |  |
| <100,000 | 1.00 (reference) |  |  | 1.00 (reference) |  |  | 1.00 (reference) |  |
| ≥100,000 | 0.54 (0.21–1.38) | 0.196 |  | 0.55 (0.23–1.34) | 0.189 |  | 1.07 (0.49–2.33) | 0.869 |
| Time interval, months |  |  |  |  |  |  |  |  |
| <1 | 1.00 (reference) |  |  | 1.00 (reference) |  |  | 1.00 (reference) |  |
| ≥1 | 0.50 (0.16–1.53) | 0.226 |  | 2.18 (0.86–5.52) | 0.101 |  | 1.41 (0.62–3.24) | 0.412 |
| Diabetes |  |  |  |  |  |  |  |  |
| No | 1.00 (reference) |  |  | 1.00 (reference) |  |  | 1.00 (reference) |  |
| Yes | 3.59 (0.79–16.23) | 0.097 |  | - | - |  | - | - |
| CKD |  |  |  |  |  |  |  |  |
| No | 1.00 (reference) |  |  | 1.00 (reference) |  |  | 1.00 (reference) |  |
| Yes | - | - |  | 6.94 (0.34–142.92) | 0.209 |  | - | - |
| HBV infection |  |  |  |  |  |  |  |  |
| No | 1.00 (reference) |  |  | 1.00 (reference) |  |  | 1.00 (reference) |  |
| Yes | 1.49 (0.34–6.53) | 0.593 |  | 0.56 (0.07–4.48) | 0.581 |  | 0.64 (0.16–2.63) | 0.536 |
| HCV infection |  |  |  |  |  |  |  |  |
| No | 1.00 (reference) |  |  | 1.00 (reference) |  |  | 1.00 (reference) |  |
| Yes | - | - |  | - | - |  | 1.70 (0.20–14.18) | 0.625 |
| Hypercholesterolemia |  |  |  |  |  |  |  |  |
| No | 1.00 (reference) |  |  | 1.00 (reference) |  |  | 1.00 (reference) |  |
| Yes | 0.92 (0.23–3.67) | 0.909 |  | - | - |  | 4.78 (0.92–24.71) | 0.062 |
| Hypertension |  |  |  |  |  |  |  |  |
| No | 1.00 (reference) |  |  | 1.00 (reference) |  |  | 1.00 (reference) |  |
| Yes | - | - |  | 5.27 (0.30–91.08) | 0.253 |  | - | - |
| Opportunistic infections |  |  |  |  |  |  |  |  |
| No | 1.00 (reference) |  |  | 1.00 (reference) |  |  | 1.00 (reference) |  |
| Yes | 1.74 (0.29–10.45) | 0.546 |  | - | - |  | 0.85 (0.11–6.48) | 0.872 |
| ART regimen (collapsed) |  |  |  |  |  |  |  |  |
| NNRTI-based | 1.00 (reference) |  |  | 1.00 (reference) |  |  | 1.00 (reference) |  |
| INSTI-based | 6.09 (2.54–14.62) | <0.001 |  | 1.76 (0.53–5.82) | 0.353 |  | - | - |
| PI-based | 1.70 (0.22–13.18) | 0.611 |  | 2.71 (0.56–13.15) | 0.215 |  | 0.67 (0.09–5.00) | 0.693 |
| **Chlamydia (τ=8.5)** | | | | | | | | |
| Marital status |  |  |  |  |  |  |  |  |
| Married or cohabiting | 1.00 (reference) |  |  | 1.00 (reference) |  |  | 1.00 (reference) |  |
| Never married | 2.29 (0.46–11.45) | 0.315 |  | 1.58 (0.44–5.64) | 0.484 |  | 2.99 (1.12–7.96) | 0.029 |
| Divorced/widowed | 0.77 (0.12–5.02) | 0.782 |  | 3.70 (0.53–25.86) | 0.188 |  | 1.47 (0.29–7.37) | 0.642 |
| Unknown | - | - |  | - | - |  | - | - |
| BMI, kg/m² |  |  |  |  |  |  |  |  |
| 18.5–24.9 | 1.00 (reference) |  |  | 1.00 (reference) |  |  | 1.00 (reference) |  |
| <18.5 | 1.01 (0.30–3.37) | 0.988 |  | 1.13 (0.22–5.87) | 0.887 |  | 1.16 (0.48–2.84) | 0.738 |
| ≥25.0 | 0.69 (0.14–3.44) | 0.648 |  | 1.96 (0.28–13.77) | 0.5 |  | 1.42 (0.45–4.49) | 0.551 |
| WBC |  |  |  |  |  |  |  |  |
| Normal | 1.00 (reference) |  |  | 1.00 (reference) |  |  | 1.00 (reference) |  |
| Abnormal | 1.09 (0.33–3.58) | 0.885 |  | 1.02 (0.28–3.76) | 0.974 |  | 1.24 (0.55–2.81) | 0.61 |
| Platelet |  |  |  |  |  |  |  |  |
| Normal | 1.00 (reference) |  |  | 1.00 (reference) |  |  | 1.00 (reference) |  |
| Abnormal | 1.60 (0.46–5.60) | 0.464 |  | 1.86 (0.49–7.05) | 0.359 |  | 1.06 (0.41–2.71) | 0.911 |
| eGFR |  |  |  |  |  |  |  |  |
| Normal | 1.00 (reference) |  |  | 1.00 (reference) |  |  | 1.00 (reference) |  |
| Abnormal | - | - |  | 1.08 (0.10–12.09) | 0.951 |  | 1.06 (0.24–4.63) | 0.936 |
| ALT |  |  |  |  |  |  |  |  |
| Normal | 1.00 (reference) |  |  | 1.00 (reference) |  |  | 1.00 (reference) |  |
| Abnormal | 0.75 (0.15–3.64) | 0.719 |  | 0.47 (0.07–3.43) | 0.459 |  | 0.83 (0.32–2.20) | 0.714 |
| AST |  |  |  |  |  |  |  |  |
| Normal | 1.00 (reference) |  |  | 1.00 (reference) |  |  | 1.00 (reference) |  |
| Abnormal | 0.61 (0.09–4.27) | 0.619 |  | - | - |  | 1.19 (0.37–3.87) | 0.77 |
| Initial CD8 count (cells/μL) |  |  |  |  |  |  |  |  |
| 500–999 | 1.00 (reference) |  |  | 1.00 (reference) |  |  | 1.00 (reference) |  |
| <500 | 2.24 (0.60–8.44) | 0.233 |  | 1.59 (0.36–6.92) | 0.537 |  | 0.35 (0.10–1.21) | 0.096 |
| ≥1000 | 0.82 (0.28–2.42) | 0.714 |  | 0.91 (0.25–3.30) | 0.887 |  | 0.96 (0.45–2.04) | 0.921 |
| Initial HIV RNA (copies/mL) |  |  |  |  |  |  |  |  |
| <100,000 | 1.00 (reference) |  |  | 1.00 (reference) |  |  | 1.00 (reference) |  |
| ≥100,000 | 1.20 (0.45–3.17) | 0.715 |  | 0.68 (0.23–2.01) | 0.488 |  | 1.72 (0.88–3.39) | 0.115 |
| Time interval, months |  |  |  |  |  |  |  |  |
| <1 | 1.00 (reference) |  |  | 1.00 (reference) |  |  | 1.00 (reference) |  |
| ≥1 | 0.40 (0.13–1.24) | 0.112 |  | 1.03 (0.33–3.26) | 0.956 |  | 1.13 (0.58–2.20) | 0.714 |
| Diabetes |  |  |  |  |  |  |  |  |
| No (ref) | 1.00 (reference) |  |  | 1.00 (reference) |  |  | 1.00 (reference) |  |
| Yes | 1.55 (0.52–4.66) | 0.432 |  | 0.50 (0.04–6.60) | 0.595 |  | 0.47 (0.06–4.04) | 0.493 |
| CKD |  |  |  |  |  |  |  |  |
| No (ref) | 1.00 (reference) |  |  | 1.00 (reference) |  |  | 1.00 (reference) |  |
| Yes | - | - |  | - | - |  | - | - |
| HBV infection |  |  |  |  |  |  |  |  |
| No | 1.00 (reference) |  |  | 1.00 (reference) |  |  | 1.00 (reference) |  |
| Yes | 1.24 (0.26–5.91) | 0.791 |  | 2.42 (0.65–9.05) | 0.189 |  | 0.62 (0.19–2.10) | 0.446 |
| HCV infection |  |  |  |  |  |  |  |  |
| No | 1.00 (reference) |  |  | 1.00 (reference) |  |  | 1.00 (reference) |  |
| Yes | - | - |  | - | - |  | 1.70 (0.37–7.83) | 0.495 |
| Hypercholesterolemia |  |  |  |  |  |  |  |  |
| No | 1.00 (reference) |  |  | 1.00 (reference) |  |  | 1.00 (reference) |  |
| Yes | 0.77 (0.26–2.27) | 0.639 |  | 6.08 (0.93–39.59) | 0.059 |  | 3.98 (1.09–14.48) | 0.036 |
| Hypertension |  |  |  |  |  |  |  |  |
| No | 1.00 (reference) |  |  | 1.00 (reference) |  |  | 1.00 (reference) |  |
| Yes | 10.02 (3.82–26.28) | <0.001 |  | 7.21 (1.22–42.46) | 0.029 |  | - | - |
| Opportunistic infections |  |  |  |  |  |  |  |  |
| No | 1.00 (reference) |  |  | 1.00 (reference) |  |  | 1.00 (reference) |  |
| Yes | 2.40 (0.37–15.38) | 0.357 |  | 2.48 (0.25–24.09) | 0.435 |  | 1.01 (0.23–4.39) | 0.993 |
| ART regimen (collapsed) |  |  |  |  |  |  |  |  |
| NNRTI-based | 1.00 (reference) |  |  | 1.00 (reference) |  |  | 1.00 (reference) |  |
| INSTI-based | 5.35 (1.83–15.64) | 0.002 |  | 3.24 (0.87–12.07) | 0.079 |  | 6.83 (2.36–19.74) | <0.001 |
| PI-based | 2.44 (0.61–9.79) | 0.208 |  | 1.69 (0.14–20.66) | 0.681 |  | 0.92 (0.21–3.98) | 0.907 |

Time windows were defined as 0–1 year, 1–3 years, and >3 years to τ (outcome-specific administrative censoring) since antiretroviral therapy (ART) initiation. Estimates were obtained from fully adjusted Fine–Gray subdistribution hazard models treating death as a competing event and are presented as subdistribution hazard ratios (sHRs) with 95% confidence intervals (CIs) and corresponding P values. Time interval refers to the duration between HIV diagnosis and ART initiation.

Abbreviations: STI, sexually transmitted infection; ART, antiretroviral therapy; BMI, body-mass index; HIV, human immunodeficiency virus; IDU, injection drug use; WBC, white blood cells; CKD, chronic kidney disease; HBV, hepatitis B virus; HCV, hepatitis C virus; HPV, human papillomavirus; HSV-2, herpes simplex virus type 2; NRTIs, nucleoside reverse transcriptase inhibitors.

**Supplementary Fig. S1** Flow diagram of participant selection and study inclusion


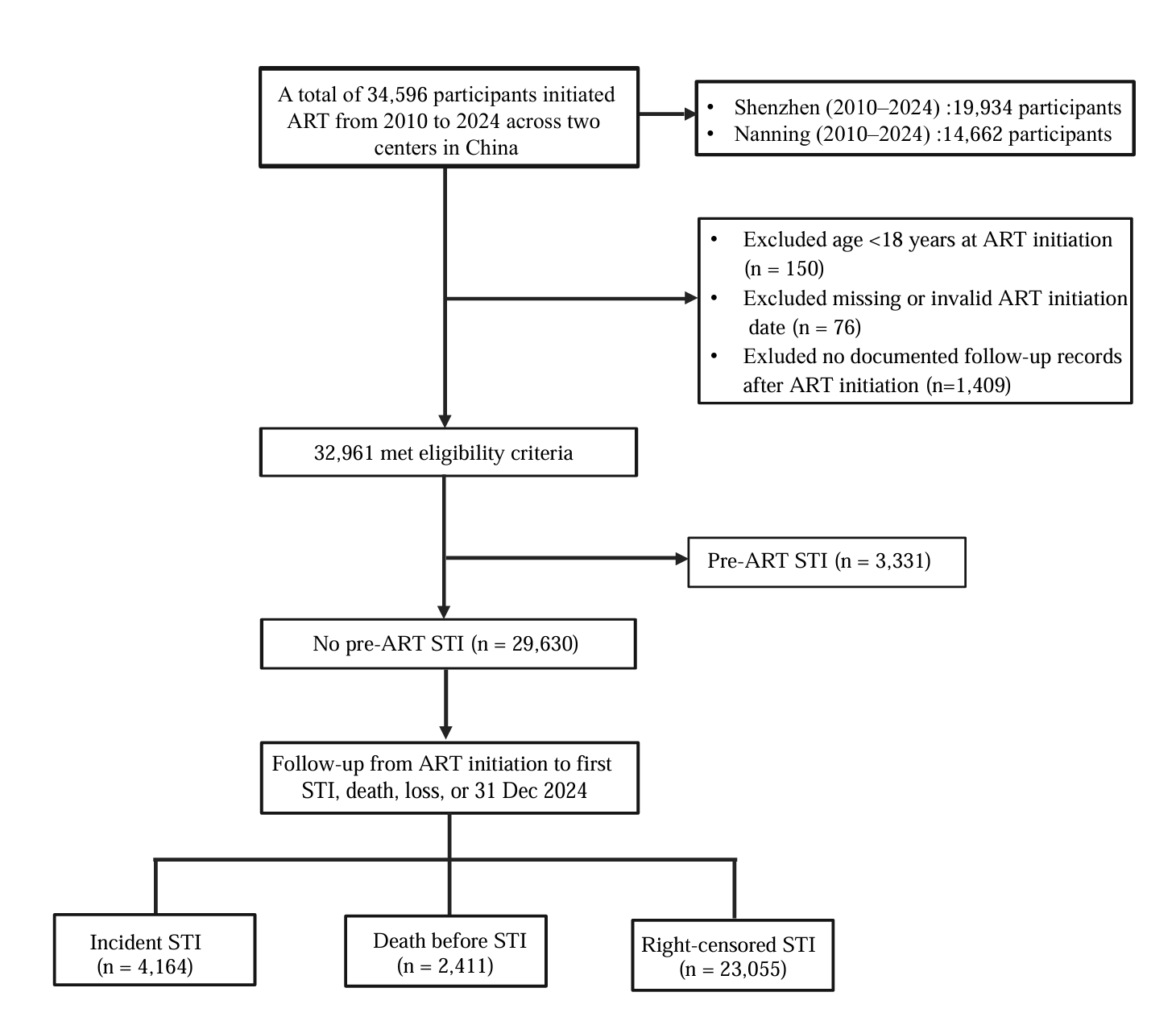


**Supplementary Fig. S2** Time-varying subdistribution hazard ratios from non-proportional Fine–Gray models for Any STIs


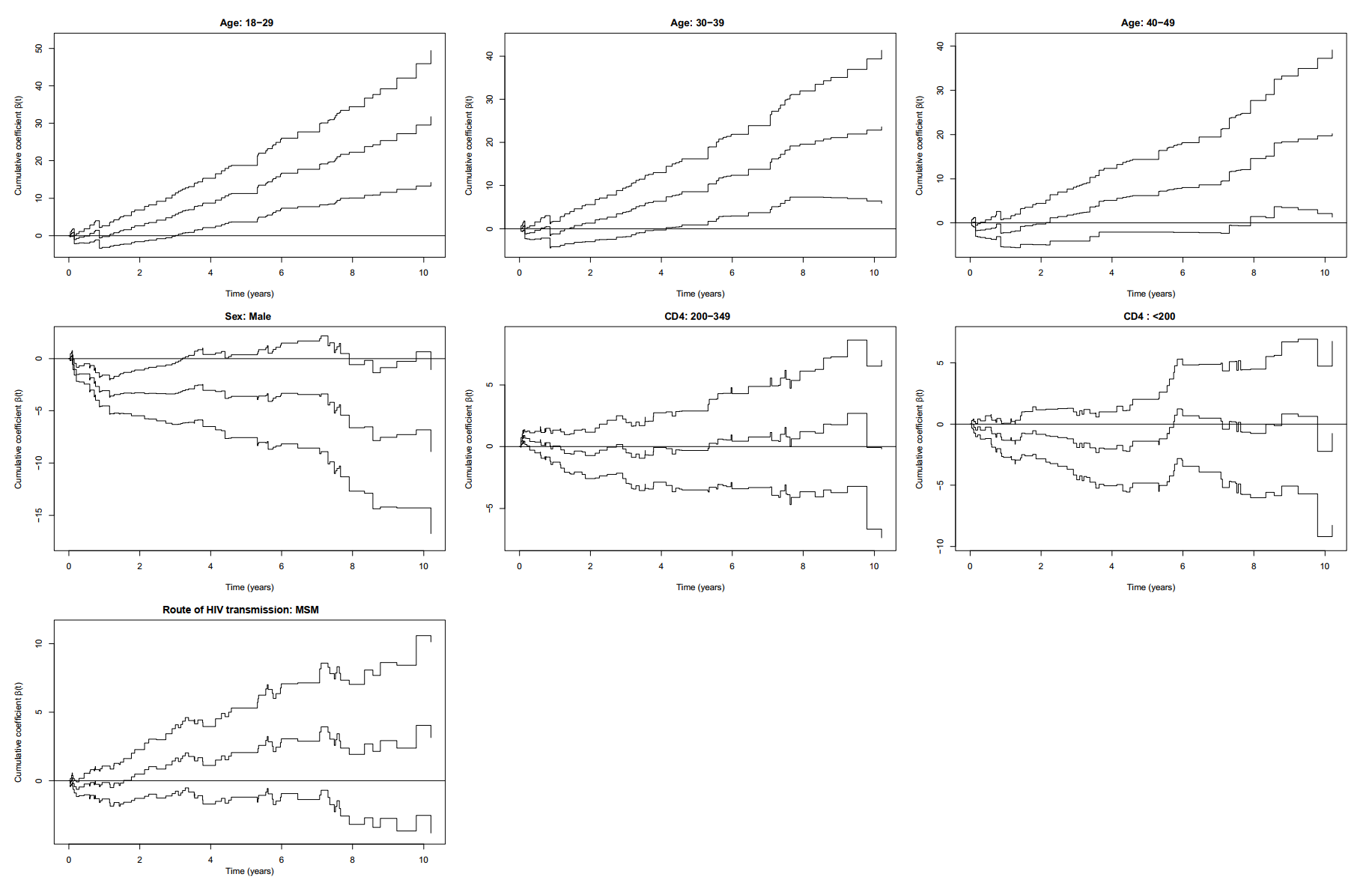


**Supplementary Fig. S3** Time-varying subdistribution hazard ratios from non-proportional Fine–Gray models for Syphilis


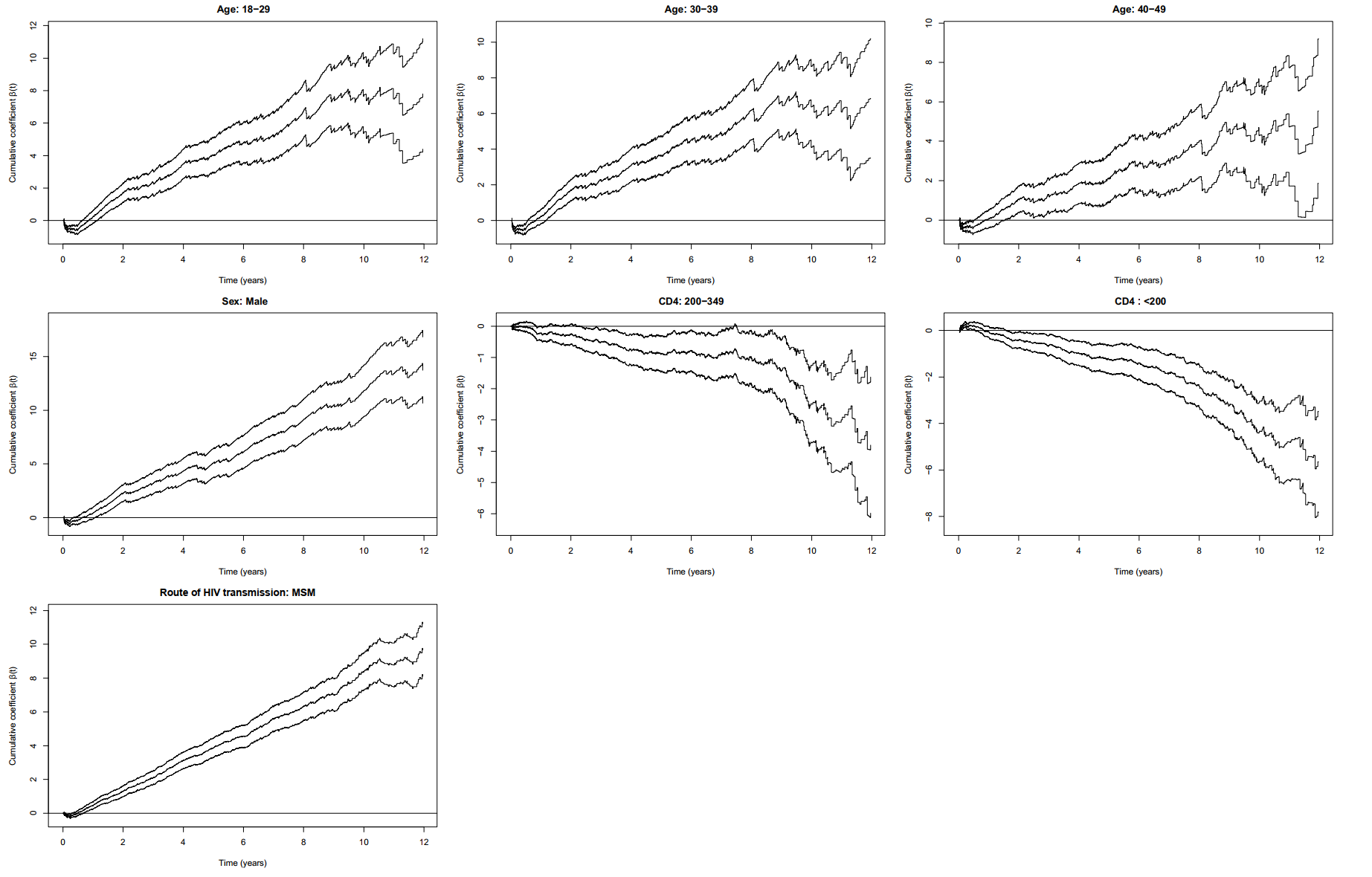


**Supplementary Fig. S4** Time-varying subdistribution hazard ratios from non-proportional Fine–Gray models for HPV


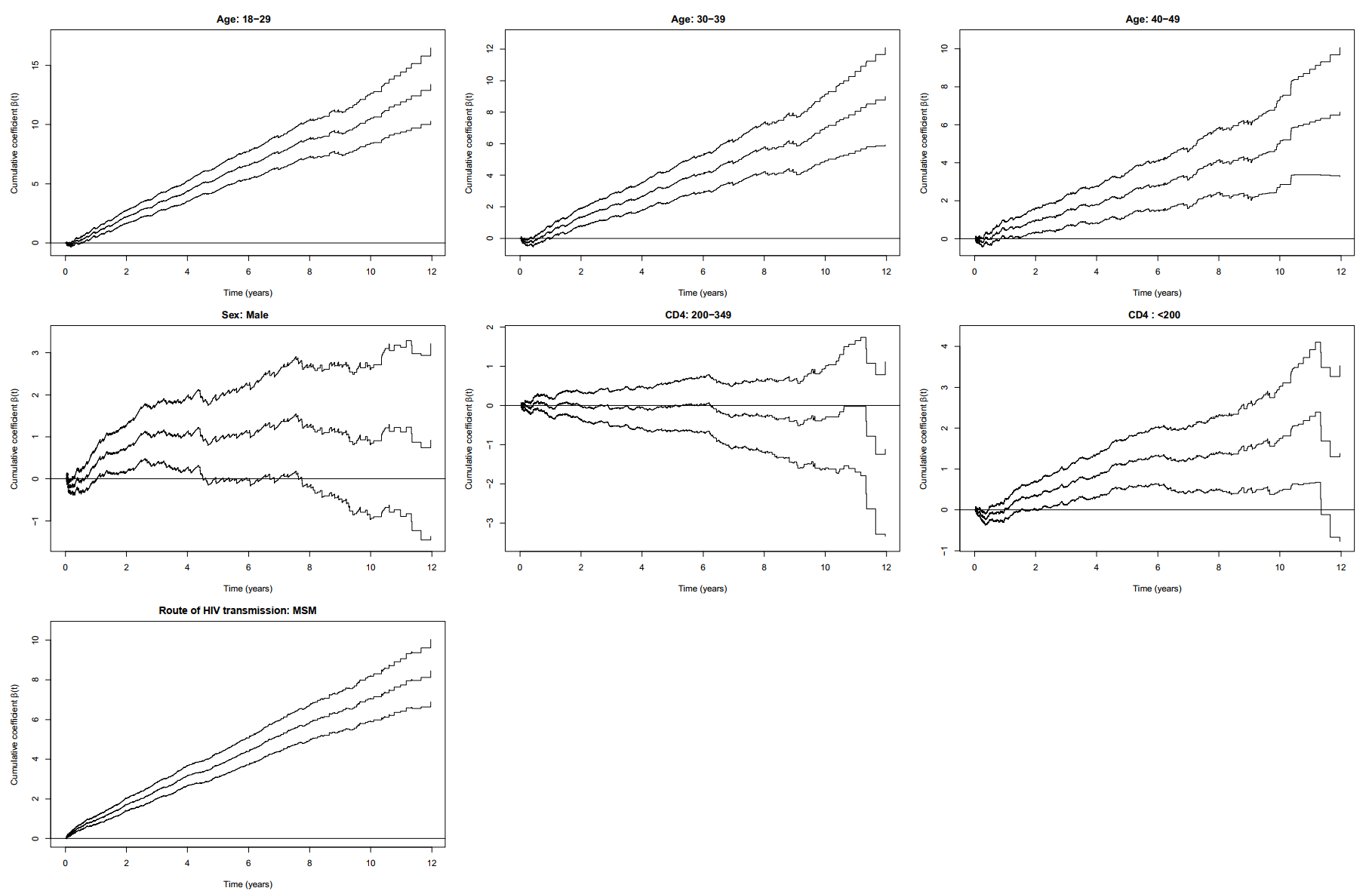


**Supplementary Fig. S5** Time-varying subdistribution hazard ratios from non-proportional Fine–Gray models for HSV-2


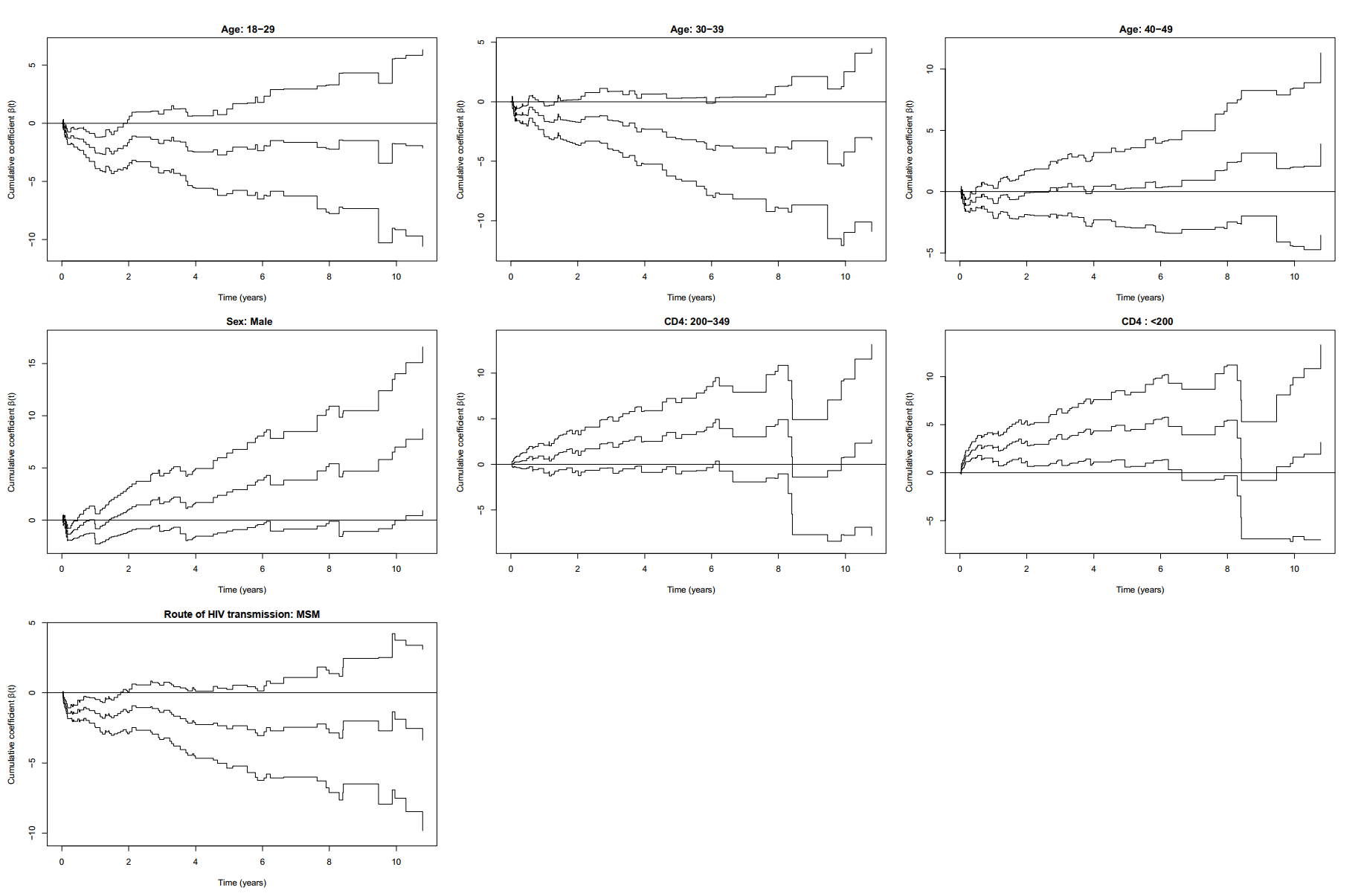


**Supplementary Fig. S6** Time-varying subdistribution hazard ratios from non-proportional Fine–Gray models for Gonorrhoea


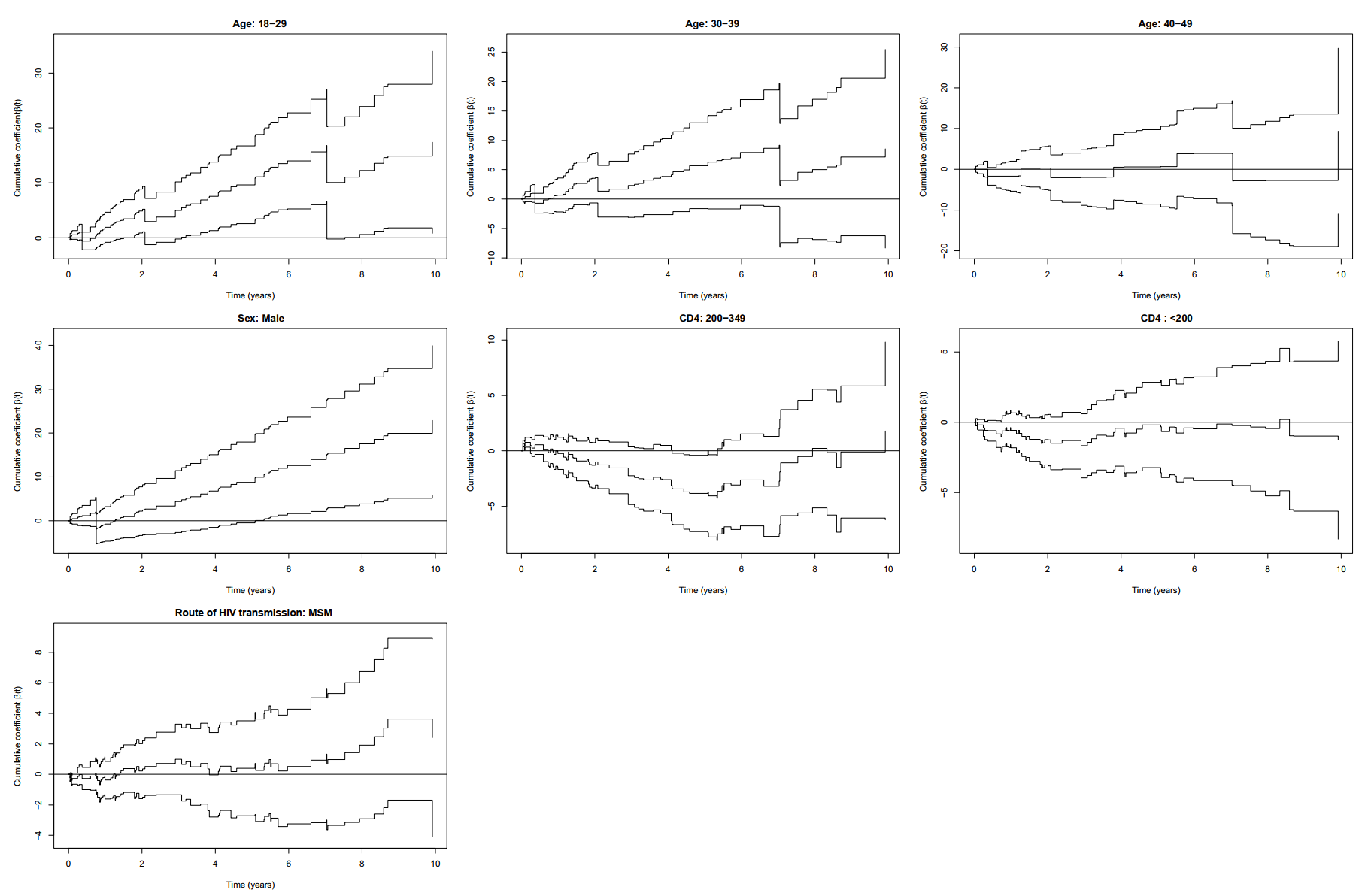


**Supplementary Fig. S7** Time-varying subdistribution hazard ratios from non-proportional Fine–Gray models for Chlamydia


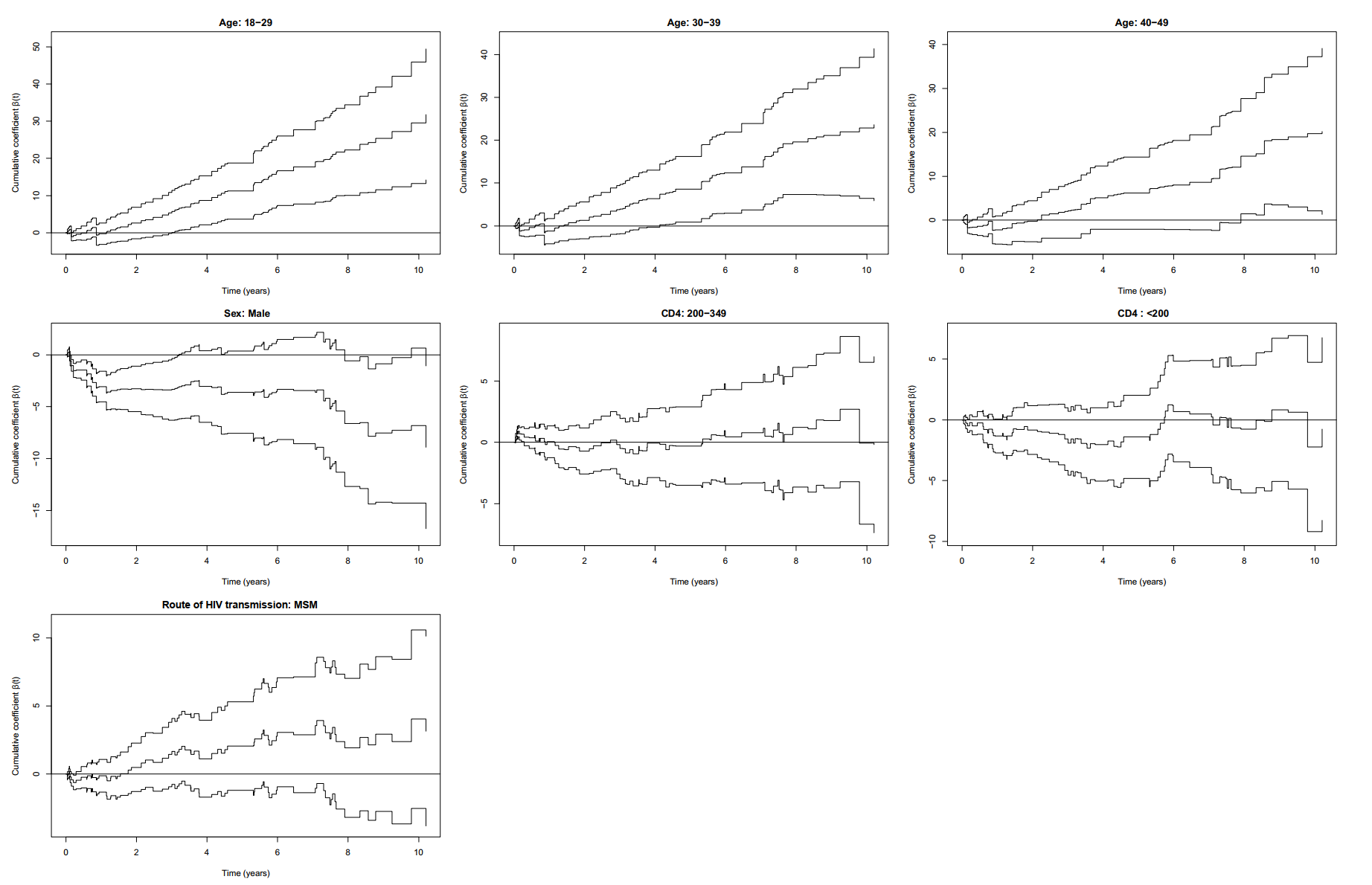


**Supplementary Fig. S8** Annual incidence rates of STIs after excluding participants with only one follow-up visit, 2010–2024.


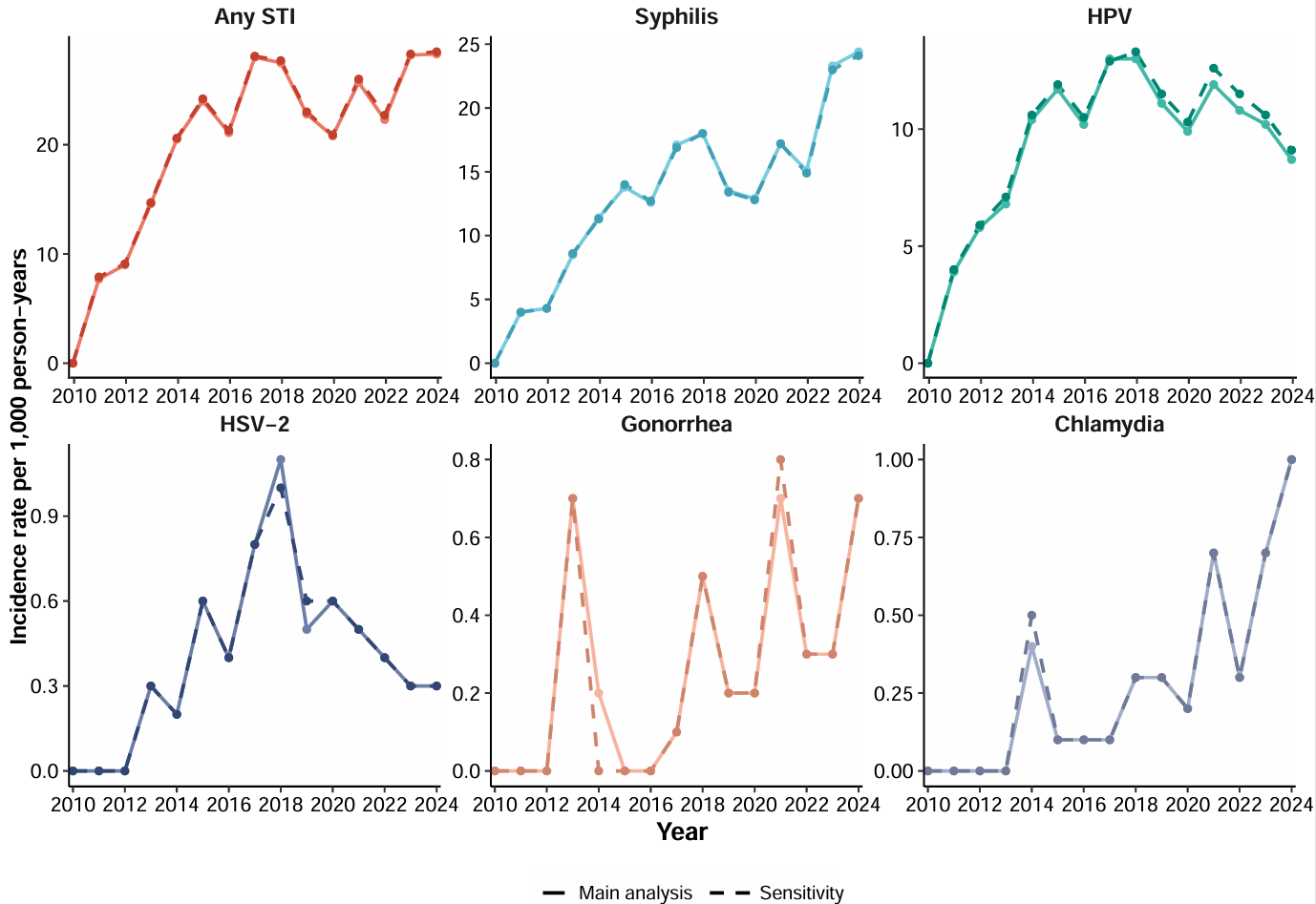


**Supplementary Fig. S9** Aalen–Johansen cumulative incidence of syphilis, HPV, HSV-2, gonorrhea, and chlamydia, stratified by sex


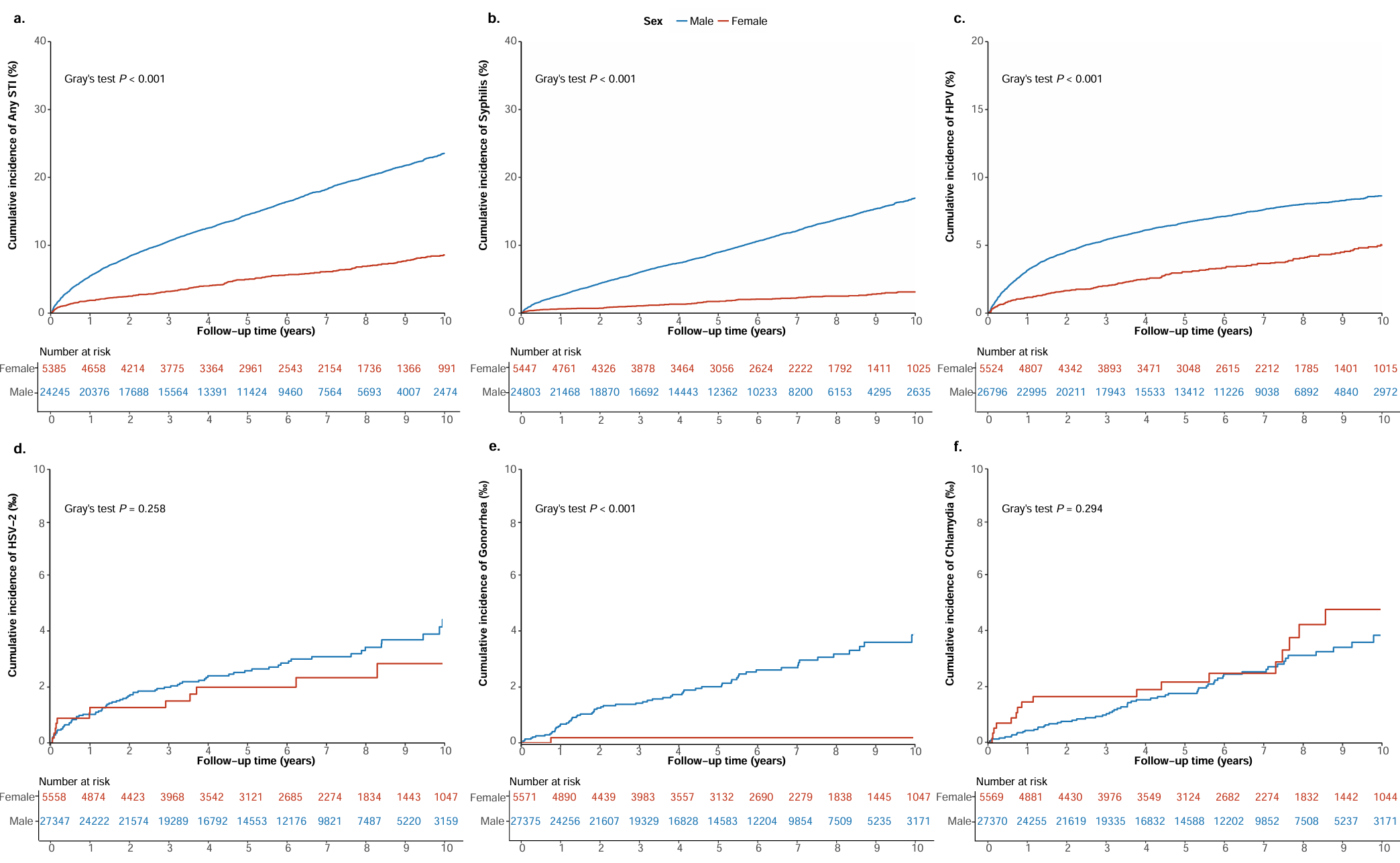


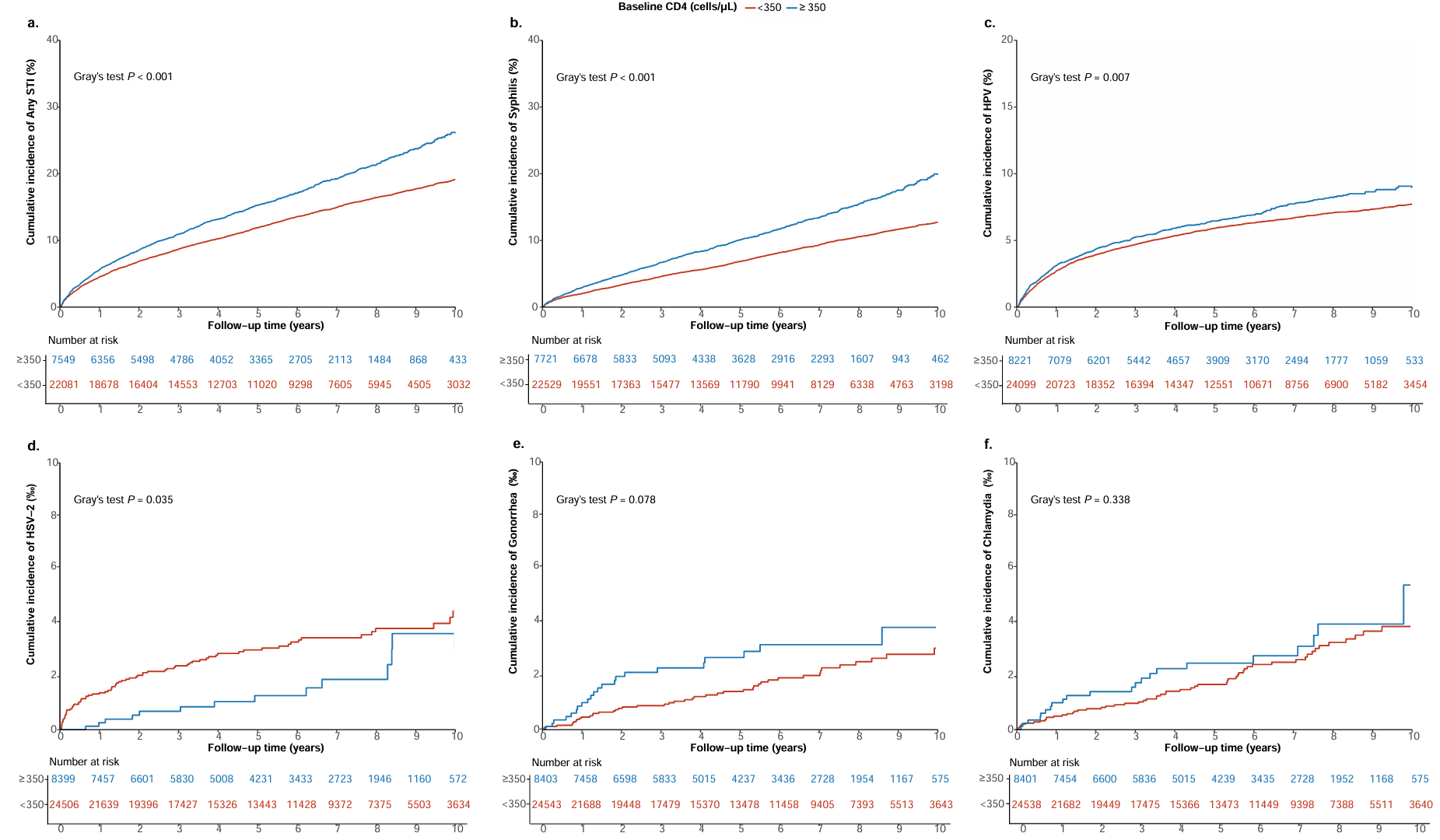
**Supplementary Fig. S10** Aalen–Johansen cumulative incidence of syphilis, HPV, HSV-2, gonorrhea, and chlamydia, stratified by CD4 group
